# Risk formulation mechanism among top global energy companies under large shocks

**DOI:** 10.1371/journal.pone.0322462

**Published:** 2025-05-23

**Authors:** Xin Qi, Tianyu Zhao

**Affiliations:** 1 Institute of Chinese Financial Studies, Southwestern University of Finance and Economics, Chengdu, China; 2 School of Finance, Shanghai University of Finance and Economics, Shanghai, China; Shanghai Jiao Tong University, CHINA

## Abstract

Taking top global energy companies as the epitome, this paper investigates the risk formulation mechanism of the international energy market under the impact of large shocks. We first use the machine learning method in (Liu and Pun, 2022) to calculate the systematic risk level - EMES - for each energy company. Then use network analysis methods to explore the internal risks due to risk comovement among top energy companies. Finally, a dynamic quantile regression model(DNQR) is used to investigate the external risks occasioned by network effects, individual company characteristics, and market environment. Our research finds that the method we use can capture the risk profile of the energy market under different major shocks. Secondly, we find that the risk contagion in the energy market exhibits geographical clustering characteristics, and certain firm-specific factors and market environmental factors of the company have a significant impact on the tail risk of the company. Our research can provide reference and guidance for risk management in the energy market.

## 1 Introduction

The global financial crisis in 2008 had a wide-ranging and profound impact on the world economy, and an integral part of which, the international energy market, inevitably suffered severe shocks. Since then, financial shocks, trade frictions, public health emergency, and geopolitical conflicts have frequently occurred, exerting huge impact on the structure and development of the global energy market. Hugeshocks are usually of suddenness and urgency [1], infusing global energy markets with great uncertainty, and are especially so since green and low-carbon transformation is widely accelerated in the energy industry. Therefore, it proves necessary to see through and describe the mechanism where such risks—in a global energy market facing great shocks—are generated, so that stability and sustainability may be restored to this critical market.

In the energy sector, large energy companies often hold a dominant market position and play a leading role in the market [2]. They are crucial in meeting the energy demands of global economic growth and rapid industrialization with global population growing continuously [3, 4]. In the 2022 Fortune Global 500 ranking, a total of 78 energy companies made the list. Top energy companies not only hold significant positions in their domestic energy markets, but are also closely linked to each other in terms of international energy business under the context of global energy market integration [5, 6]. Therefore, the stable operation of these energy companies is a prerequisite to the smooth functioning of the global energy market.

However, evidence suggests that large energy companies have been facing significant business challenges and financial pressures under the trend of low-carbon transition [7, 8]. The outbreak of the COVID-19 pandemic and the Russia–Ukraine conflict in recent years have further rendered the uncertainty in energy company operations. In the face of the transformation risks in the global energy market, large shocks can lead to more complex risk correlations among large energy companies. Therefore, exploring the risk generation mechanism among top global energy companies during major shocks is crucial for enhancing their risk management capabilities and for regulatory authorities to mitigate energy market risks.

The financialization of the energy sector has become a topic of concern for scholars in recent years, a and the integration of the energy market, especially at the regional level, is particularly evident [9]. The enhanced interconnectedness of energy markets and the potential exposure to various risks make it necessary to consider additional market environment and market structure factors outside the energy market when predicting the level of risk in energy firms.With the emergence of big data and the development of data science in recent years, machine learning has been more widely applied in various fields of society. Due to its precise pattern recognition and predictive capabilities, machine learning is also expanding its application scale in the financial field, especially in the early warning and prediction of financial risks. For example, the United States has long been the center of the global economy, and the monetary policy of the Federal Reserve becomes a matter of concern to the whole world when a large shock occurs globally.In fact, the Federal Reserve’s monetary policy does have a significant impact on the energy market. Reference [10] studied whether and to what extent the Fed’s conventional and unconventional monetary policies affect energy prices, and found that unexpected monetary policy has an important and significant economic impact on energy prices, especially oil price, [11] suggests that positive monetary policy shocks can raise oil prices and that US monetary policy has unexpected consequences for energy markets, [12]present a strong link between monetary policy uncertainty (MPU) and oil price futures, and [13] find that U.S. monetary policy is unfavorable for global green investment in both the short and long term. However, the significant impact of US monetary policy has not been taken into account in previous papers using traditional methods to measure the systemic risk of energy companies in the energy market.

In contrast, machine learning algorithms, which do not make any predetermined assumptions about the functional form of equations, the interactions between the variables, and the statistical distribution of the parameters [14], are a method of risk measurement that can take into account a combination of multiple market environment factors, including the Federal Reserve’s monetary policy. It is also necessary to incorporate market structure factors into the risk prediction of top energy companies, considering that top energy companies are affected by both regional and international financial markets.

At the same time, in order to understand the interconnectedness among top global energy companies at the overall system level, it is necessary to examine the measurement, degree, and asymmetry of systemic risks. The spillover effects among the risk networks of global energy companies have brought increasing uncertainty to energy security [15], and connectedness among top energy firms is an important role in understanding systemic risk, as it refers to the financial trouble of one energy company leading to financial failure of other energy companies through business dealings with different companies. This is due to the fact that the scale of energy production in individual countries cannot meet the increasing domestic demand, and firms engaged in upstream and downstream energy-related activities are being globally connected, and the shift from energy independence to energy interdependence has led to the integration of the world’s energy markets [16]. This trend of integration and financialization, together, has led to a networked and complex risk contagion in energy markets, making it crucial for both market managers and investors to accurately measure systemic risks in the international energy market [17].

In this paper, we analyze how major shocks affect risk generation in the global energy market from both internal and external perspectives. We refer to [18, 19] for the selection of great shock events:the global financial crisis, the European debt crisis, the Brexit Referendum, the US–China trade disputes, and the COVID-19 pandemic. They select relevant events by the criteria of significant increase in corresponding risk exposure indicators and international impact, and verify that these events meet the characteristics of major shock events. Our study has yielded some interesting findings. Firstly, we observe that top energy companies experience varying degrees of increase in their systemic risk levels during major shocks. Moving on to the analysis of individual energy companies, we identify Petro China Co Ltd as a key player in the global energy market risk network based on the systemic risk scores. We also confirm the significant positions of Royal Dutch Shell plc, Total Energies SE, Chevron Corp, and Exxon Mobil Corp in the risk network, which aligns with the findings of [2, 4]. Finally, employing the DNQR model, we find that debt leverage levels, foreign exchange market factors, and investor sentiment significantly influence the extreme left-tail risk of energy companies.

This paper makes three main contributions. Firstly, we compare and analyze the impacts of five representative large shocks—the global financial crisis, the European debt crisis, the Brexit Referendum, the US–China trade disputes and the COVID-19 pandemic—on energy market risks, enriching the existing literature. Secondly, the paper comprehensively examines the risk generation mechanisms of top energy companies from both internal and external perspectives. Internally, the risk generation in the energy market mainly arises from the risk co-movements among top energy companies and their own systemic risk accumulation. We employ a systemic risk measurement approach incorporating machine learning algorithms to provide a more exhaustive characterization of the systemic risks of energy companies. As regard external factors, the paper investigates the influence of company characteristics, cross-market contagion, investor sentiment, and policy uncertainty on the returns of top energy companies, particularly extreme returns, and discovers those factors that greatly affect tail risks. Lastly, the paper identifies the key energy companies that play a crucial role in the risk correlation network of the global energy market during large shocks, complementing the research conducted by [2, 4]. The research findings on risk generation in the global energy market during large shocks provide valuable investment insights and policy implications for energy company operators, energy market investors, and government regulatory authorities.

## 2 Literature review

### 2.1 Risk spillovers of energy markets

Extensive research has been conducted on the risk spillover of energy markets. Numerous studies have discussed the risk spillover among major energy commodities [20, 21]. Reference [22] combine the ensemble empirical mode decomposition (EEMD) and [23] connectedness network framework to study the information spillover between oil and gas markets. The conclusion supports the one-way information spillover from oil to gas, which is dynamic and unstable. Reference [24] find that the crude oil market is the main recipient of volatility spillovers between crude oil and other asset markets, while the stock market is the main contributor to volatility spillovers. Reference [25] focus on the volatility spillover among the four major crude oil markets (WTI, Brent, Oman, Tapis). Their research reveals a gradual increase in volatility spillover, indicating growing interconnectedness among these markets, [26] also found this connection between Shanghai futures and the US crude oil futures market. In recent years, energy markets in major countries have become increasingly linked [6]. Reference [27] study the connectedness between energy stock indices of oil-exporting and oil-importing countries during the COVID-19 pandemic. They construct a national energy risk network and quantified the risk transmission and reception between different stock indices. The United States, France, Italy, Canada, Norway, and Spain are identified as the major shock transmitters. Reference [28] explore the time-varying risk linkage between the Chinese energy market and the international energy market, and find the increasing influence of Chinese energy market price volatility on international energy price volatility. Furthermore, in the context of global climate change and low-carbon transition, the risk spillovers from climate change, climate policy and green energy have become a hot topic recently [29–32]. Overall, there is a large body of research in the existing literature on the subject of risk spillovers between energy markets, but these studies have been limited to risk contagion between energy markets in individual countries, between oil and gas markets, and between energy commodities and other commodities. Considering the increasing trend of international energy market integration, developing countries are increasingly participating in international energy trading, and the energy industry is not limited to the oil and gas industry, it is necessary to conduct further research on the spillover effects of energy market risks, including those in various regions around the world.

Given the increasing influence of large energy companies in the global energy system, scholars have shifted their focus from energy commodities to energy companies in recent research. Reference [3] analyze the total average connectedness and pairwise spillovers among the 20 largest publicly listed oil companies on the New York Stock Exchange, considering both static and dynamic perspectives. Reference [33] study the interdependence between WTI and the returns of publicly listed energy companies in the United States. The results indicate that the US energy sector is a net contributor to WTI price changes, with the oil and gas industry dominating other industries. However, these studies are limited to traditional energy sectors such as oil and gas, and recent research has expanded the scope to encompass a more comprehensive range of energy industries. Reference [34] measure the risk spillover among 20 high-weight new energy companies in the NEX index. They find asymmetric effects in the information spillover mechanism among global new energy companies, and identify some companies as important information transmitters in the network. Reference [4] study the risk contagion among the top 20 energy companies in the Platts global energy company rankings. They construct a systemic risk index for the energy market system and find that the dynamic changes in risk are mainly driven by fluctuations in the US stock market and investor sentiment. Subsequently, [2] expand the research sample to the top 100 global energy companies and use a multilayer network approach to explore the risk contagion relationships among these energy companies. The study further divides the companies into three major regions based on geographic location to investigate the risk spillover within the regions. Overall, research on risk spillover among energy companies is still in its initial stages, and further studies are needed to understand the risk generation mechanisms within energy company networks. Although numerous researchers starting to study the risk contagion network of energy companies, there are obvious limitations in their research samples, which mostly focus on a few top energy companies in developed markets. Considering that developing countries such as China, Brazil, India, and Russia are becoming increasingly active in the international energy market, it is necessary to incorporate more energy companies from around the world in the research example. After considering the availability of data, our study included 192 energy companies from 33 countries in Platt’s global energy company ranking, which makes our study about international energy market more comprehensive and representative.

A considerable portion of the literature on energy market risk spillover has applied different types of network analysis methods to construct risk spillover networks [35–38]. In the study of tail risk spillovers among market participants, some studies have employed network analysis methods [39, 40]. To better analyze the factors influencing tail risk, some researchers extend the network analysis approach by introducing the network quantile regression model [41–43]. However, it should be noted that, as far as our knowledge goes, the network quantile regression method has not been applied to study the factors influencing tail risk among energy companies. In this study, we employ the DNQR method proposed by [43]. This method not only takes into account firm-specific characteristics and market environmental factors but also incorporates the influence of other energy companies’ returns that are connected through a risk comovement network in both contemporaneous and lagged periods, thus enabling a comprehensive exploration of the factors influencing the tail returns of energy companies under major shocks.

There is evidence indicating that specific major events, such as geopolitical conflicts and financial crises, can have a significant impact on the volatility of energy market prices [19, 36, 44–48]. It is worth noting that current research predominantly concentrates on the effects of single major shocks, with relatively limited studies examining the impacts of multiple major shocks on the energy market and investigating the underlying mechanisms of risk generation. In this context, our article primarily delves into the risk generation mechanisms among top global energy companies under major shocks, thereby making a valuable contribution to the existing research on risk prevention within the energy market.

### 2.2 Systemic risk measurement

The characteristics and measurement of systemic risk in energy markets have been a focal topic of interest among scholars [2, 49–53]. In this regard, [54] propose Conditional Value-at-Risk (CoVaR) as a measure to reveal the tail risk level faced by financial institutions during systemic risk events. The difference between CoVaRs in extreme and normal states, known as △CoVaR, is used to quantify the systemic risk of financial institutions. Building on this idea, some studies have employed △CoVaR to measure the systemic risk in energy markets. Reference [55] analyze the dynamic evolution of systemic risk measured by △CoVaR for oil and gas companies. They find that both company’s losses and size contribute to systemic risk. Reference [56] employ △CoVaR to measure the systemic risk contribution of commodities to financial markets and find a stronger risk contagion effect during the period of 2017–2019. Additionally, [57] propose the concept of marginal expected shortfall (MES), which captures the marginal contribution of financial institutions to systemic risk. This measure has also been utilized to quantify the contribution of energy prices to systemic risk. For example, [58] and [59] have both calculated the MES measure in addition to △CoVaR, and further analyze the impact of oil prices on systemic risk. The calculation of CoVaR has the advantage of considering macro-state covariates. In this paper, we calculate the EMES proposed by [60]. This measure incorporates the advantages of CoVaR into the calculation of MES for energy companies by using a dynamic systemic event threshold that considers the influence of macroeconomic variables. Furthermore, the aforementioned indicators only consider the direct risk linkages between systemic events and energy companies, without taking into account the cascading effects of systemic risk. Reference [61] conduct a study on the systemic risk of European financial firms and find a stratified dependency between European firms’ return and the return of country, European and the world. By incorporating machine learning algorithms, the EMES indicator in this paper takes into account the hierarchical structure of return dependency among the US, host country, and firms, providing a more comprehensive characterization of systemic risk levels in energy companies.

### 2.3 Machine learning algorithms in energy study

Machine learning algorithms have long been applied in the study of energy, except in the field of energy engineering [62–64], but also in a large number of applications for oil and electricity price forecasting [18, 65–71]. Reference [14] argued that the advantage of machine learning over traditional econometric models such as ARIMA and GARCH is that machine learning algorithms can manage large amounts of structured and unstructured data and make decisions or predictions quickly.Beyond the aforementioned application areas, [72] attempted to predict the volatility of crude oil and natural gas prices using different machine learning algorithms, while [73] used machine learning algorithms to predict the impact of crude oil and other countries’ stock markets on the Saudi stock market. In the field of predicting systematic risk levels in energy companies, machine learning algorithms have not yet been effectively applied.

## 3 Methodology

First, we use a two-step supervised machine learning method Adaboost to calculate each energy company’s systemic risk level measured by EMES. Then, we build risk correlation networks among top energy companies during the five major shocks, and calculate the capitalization-based systemic risk score for each energy company. Finally, we use the DNQR method to explore the contribution of a group of risk factors to the tail risks of top energy companies in different periods.The above implementations of EMES, risk correlation networks, systemic risk score and DNQR are available in the attached code.

### 3.1 Estimating EMES via machine learning method

Following [60], we first calculate the EMES which is regarded as a systemic risk index for each energy company. This index introduces machine learning algorithms and provides an enhanced version MES. Compared with MES, EMES considers the dynamic market conditions, the close linkages between macroeconomics and financial markets, and the hierarchical dependence of the U.S. market, the market of the country where the company is located, and the company’s individual stock returns. Accordingly, EMES is designed to capture the systemic risk exposure of individual energy companies under extreme market conditions with the potential to disrupt the entire financial system, incorporating the effects of the aforementioned factors.

Firstly, it is essential to rigorously define extreme market conditions in order to identify periods of heightened market stress, which are often associated with the potential emergence of systemic risk and may pose significant threats to the stability of the financial system. Some studies have confirmed that the US stock market can lead the information flow of global stock markets and play a key role in influencing the global energy systemic risk pattern [4, 74]. Therefore, we define the S&P 500 index not exceeding its Value-at-Risk (VaR) as a systemic risk event. Specifically, like [54], systemic risk event between period *t* + 1 and *t* + *h*, denoted as *SE*_*t* + 1:*t* + *h*_, is defined by:


$$SE_{t+1:t+h}=\left\{R_{t+1:t+h}^{\text{sys }}⩽{\mathrm{VaR}}_{t+1:t+h}^{\text{sys}}\right\},$$

where $R_{t+1:t+h}^{\text{sys }}$ and ${\mathrm{VaR}}_{t+1:t+h}^{\text{sys}}$ denote the return and VaR of the S&P 500 between period *t* + 1 and *t* + *h*, respectively. The VaR can be estimated using quantile regression approach with some macro-economic variables or systemic risk factors as explanatory variables. In this context, the quantile regression model is employed to forecast VaR based on prevailing economic conditions. By incorporating key macroeconomic factors such as market volatility and interest rates, the model enhances the accuracy of estimating the probability of extreme market downturns, thereby improving the identification of potential systemic risk events.

Let *M*_*t*_ be the *p*-dimensional vector of macro-economic variables at time *t*. Consider the following quantile regression at α-th quantile level, and in this paper, the value of α is set to 0.05:


$$R_{s+1:s+h}^{\text{sys }}=\beta_{0}^{\alpha ,\text{sys }}+\beta_{1}^{\alpha ,\text{sys}⊤}M_{s}+\varepsilon_{s+1:s+h}^{\alpha ,\text{sys }},\;s=t-h-T+1,\ldots ,t-h.$$

Denote the coefficient estimates as ${\hat{\beta }}_{0}^{\alpha ,\text{sys }}$ and ${\hat{\beta }}_{1}^{\alpha ,\text{sys}}$, then the predicted VaR is


V1VaR^t+1:t+hα,sys=β^0α,sys +β^1α,sys⊤Ms.

The macro-economic variables employed in our quantile regression include the US market volatility, the real estate excess return, the credit spread change, the short-term TED spread, the term spread change, and the 3-month yield change. Following [60], the macro-economic variables are collected on daily basis.

After introducing the definition of the systemic risk event, we use machine learning methods to estimate EMES. Based on the idea of [61], the risks of energy companies exhibit a hierarchical structure, where the returns of each energy company depend on its country’s market returns and the US stock market returns. Note that there is a chain of influence from the returns of the US stock market to the returns of energy companies, where each preceding returns are affected by the subsequent returns in the chain. Based on this framework, the global energy market can be modeled as a multi-layered hierarchical dependence network, with the US stock market at the top, exerting systemic influence on a global scale. Fluctuations in the US market first affect the stock indices of specific countries or regions, which in turn propagate down to the energy companies operating within those markets.

Specifically, for the majority of energy companies, we consider three hierarchical dependence structures: US (i.e. sys) → Country Stock Index → Energy Companies. For US energy companies, given their direct and strong linkage to the US stock market, the dependency structure is simplified to two levels: US → Energy Companies. In contrast, for regions such as Hong Kong and Taiwan, the dependency structure is more complex due to the interplay between regional and domestic market indices. Accordingly, we establish the four hierarchical dependence structures: US → CSI300 Index → Hang Seng Index → Energy Companies, and US → CSI300 Index → TSEC weighted index → Energy Companies, respectively.

The transmission mechanisms of systemic risk often involve complex nonlinear dependencies, particularly within multi-layered financial systems, where directly modeling these relationships can be challenging. To address this, we employ a two-step supervised learning approach that captures the hierarchical structure of systemic risk events and the interdependencies of returns across different levels, thereby enabling an effective estimation of EMES. In general, we assume that there are *m* different layers of returns affecting the returns of the *j*-th firm for j=1,…,N. For all firms, the first layer of returns represents the US market returns, and the other *m*–1 layers of returns are the returns of intermediary layers. Let $R_{t}^{H_{i}}$ and $R_{t+1:t+h}^{H_{i}}$ be returns of the *i*-th hidden layer at time *t* and between period [t:t+h], respectively, for i=2,…,m. When $2⩽i_{1}<i_{2}⩽m$, risks are propagated from the *i*_1_-th layer to the *i*_2_-th layer. Furthermore, let $M_{t}^{H_{i}}$ be the *p*_*i*_-dimensional vector of macro-economic variables at time *t* for the *i*-th layer, and $C_{t}^{j}$ be the *k*_*j*_-dimensional vector of characteristics for firm j∈{1,…,N} at time *t*. The covariates for predicting MES are defined as follows: (i) for i=2,…,m, ${𝕏}_{t}^{H_{i}}(R_{t+1:t+h}^{\text{sys }})=(M_{t}^{H_{i}},M_{t}^{H_{i}}$ · $1_{SE_{t+1:t+h}})$, where $1_{SE_{t+1:t+h}}$ is an indicator function defined on *SE*_*t* + 1:*t* + *h*_, and $M_{t}^{H_{i}}\cdot 1_{SE_{t+1:t+h}}$ is used to model the potential structural break; (ii) for j=1,…,N, ${𝕏}_{t}^{j}(R_{t+1:t+h}^{\text{sys }})=(M_{t}^{H_{m}},C_{t}^{j},M_{t}^{H_{m}}\cdot 1_{SE_{t+1:t+h}},C_{t}^{j}\cdot 1_{SE_{t+1:t+h}})$.

According to the introduced symbolic system mentioned above, we model the hierarchical dependence structure of returns as follows:


$$\left(R_{s}^{H_{2}},R_{s+1:s+h}^{\text{sys}},R_{s}^{\text{sys}},{𝕏}_{t}^{H_{2}}(R_{s+1:s+h}^{\text{sys}})\right)\mapsto {ℳ}^{H_{2}}\mapsto R_{s+1:s+h}^{H_{2}}$$

$$\left(R_{s}^{H_{i}},R_{s+1:s+h}^{H_{i-1}},R_{s}^{H_{i-1}},\ldots ,R_{s+1:s+h}^{H_{2}},R_{s}^{H_{2}},R_{s+1:s+h}^{\text{sys}},R_{s}^{\text{sys}},{𝕏}_{t}^{H_{i}}(R_{s+1:s+h}^{\text{sys}})\right)$$

$$\mapsto {ℳ}^{H_{i}}\mapsto R_{s+1:s+h}^{H_{i}}$$

$$\left(R_{s}^{j},R_{s+1:s+h}^{H_{m}},R_{s}^{H_{m}},\ldots ,R_{s+1:s+h}^{H_{2}},R_{s}^{H_{2}},R_{s+1:s+h}^{\text{sys}},R_{s}^{\text{sys}},{𝕏}_{t}^{j}(R_{s+1:s+h}^{\text{sys}})\right)$$

$$\mapsto {ℳ}^{j}\mapsto R_{s+1:s+h}^{j}$$

where $R_{s+1:s+h}^{H_{2}}$ represents the returns of the 2th layer, typically denoting the returns of the national stock market index over the period from *s*  +  1 to *s*  +  *h*. These returns depends on the returns of the 2*th* layer level the at time *s*, denoted as $R_{s}^{H_{2}}$, the returns of the systemic market (in this case, the US stock market) index over the period from *s* + 1 to *s* + *h*, represented by $R_{s+1:s+h}^{\text{sys }}$, the systemic market return at time *s*, denoted as $R_{s}^{\text{sys }}$, and the covariates ${𝕏}_{t}^{H_{2}}(R_{s+1:s+h}^{\text{sys }})$ that are related to the systemic market returns. Additionally, the hierarchical dependence structure of the returns at the *i*th layer, denoted as $R_{s+1:s+h}^{H_{i}}$, and the returns of energy company *j*, represented by $R_{s+1:s+h}^{j}$ is similarly modeled using the same approach.

Besides, for i=2,…,m and j=1,…,N, ${ℳ}^{H_{i}}$ and ${ℳ}^{j}$ are nonlinear mappings from inputs to outputs. These mappings can be learned by supervised learning approaches. Denote the learned mappings as ${\hat{ℳ}}^{H_{i}}$ and ${\hat{ℳ}}^{j}$, then the predicted EMESs for the intermediary levels and the firms at time *t* can be calculated as follows:


$${\hat{\text{EMES}}}_{t+1:t+h}^{H_{2}}={\hat{ℳ}}_{t}^{H_{2}}\left(R_{t}^{H_{2}},{\hat{\text{VaR}}}_{t+1:t+h}^{\alpha ,\text{sys}},R_{t}^{\text{sys}},{𝕏}_{t}^{H_{2}}({\hat{\text{VaR}}}_{t+1:t+h}^{\alpha ,\text{sys}})\right),$$

$${\hat{\text{EMES}}}_{t+1:t+h}^{H_{i}}={\hat{ℳ}}_{t}^{H_{i}}(R_{t}^{H_{i}},{\hat{\text{EMES}}}_{t+1:t+h}^{H_{i-1}},R_{t}^{H_{i-1}},\ldots ,{\hat{\text{EMES}}}_{t+1:t+h}^{H_{2}},R_{t}^{H_{2}},$$

$${\hat{\text{VaR}}}_{t+1:t+h}^{\alpha ,\text{sys}},R_{t}^{\text{sys}},{𝕏}_{t}^{H_{i}}({\hat{\text{VaR}}}_{t+1:t+h}^{\alpha ,\text{sys}})),$$

$${\hat{\text{EMES}}}_{t+1:t+h}^{j}={\hat{ℳ}}_{t}^{j}(R_{t}^{j},{\hat{\text{EMES}}}_{t+1:t+h}^{H_{m}},R_{t}^{H_{m}},\ldots ,{\hat{\text{EMES}}}_{t+1:t+h}^{H_{2}},R_{t}^{H_{2}},$$

$${\hat{\text{VaR}}}_{t+1:t+h}^{\alpha ,\text{sys}},R_{t}^{\text{sys}},{𝕏}_{t}^{j}({\hat{\text{VaR}}}_{t+1:t+h}^{\alpha ,\text{sys}})).$$

In this context, ${\hat{\text{EMES}}}_{t+1:t+h}^{H_{2}}$ represents the predicted EMES indicator of the national stock market, which is estimated based on the following factors: the returns of the 2th layer at time *t*, denoted as $R_{t}^{H_{2}}$, the Value-at-Risk (VaR) of the systemic market over the period from *t* + 1 to *t* + *h*, denoted as ${\hat{\text{VaR}}}_{t+1:t+h}^{\alpha ,\text{sys}}$, the returns of the systemic market index at time *t*, denoted as $R_{t}^{\text{sys}}$, and the covariates associated with systemic risk, denoted as ${𝕏}_{t}^{H_{2}}({\hat{\text{VaR}}}_{t+1:t+h}^{\alpha ,\text{sys}})$. Similarly, ${\hat{\text{EMES}}}_{t+1:t+h}^{H_{i}}$ and ${\hat{\text{EMES}}}_{t+1:t+h}^{j}$ represent the systemic risk indices for regional stock markets and energy companies, respectively. When estimating the EMES for the next hierarchical level, the EMES from the previous level are utilized, thereby reflecting the hierarchical dependence structure in the estimation process.

For the US firms, since the prediction relies solely on the systemic risk of the US market, the predicted EMESs are:


$${\hat{\text{EMES}}}_{t+1:t+h}^{j}={\hat{ℳ}}_{t}^{j}\left(R_{t}^{j},{\hat{\text{VaR}}}_{t+1:t+h}^{\alpha ,\text{sys}},R_{t}^{\text{sys }},{𝕏}_{t}^{j}({\hat{\text{VaR}}}_{t+1:t+h}^{\alpha ,\text{sys}})\right).$$

In this paper, we utilize the AdaBoost method for estimation, a technique that has been demonstrated to achieve the highest overall performance in the comparative assessment conducted by [60]. Due to limited available data, the only macro-economic variable $M_{t}^{H_{i}}$ of a hidden layer, which is a country or region in our case, is the market volatility. The volatility is calculated by the 22-day rolling standard deviation of the market return. For energy companies from Hong Kong, we also consider the impact of HIBOR. And the firm’s characteristic $C_{t}^{j}$ is the daily trading volume of its stock. In addition, *h* is set to 21, corresponding to one month.

### 3.2 Risk relationship network analyses and systemic risk score

Taking the EMES as our risk profile, we calculate the similarity matrix of energy companies’ EMES over a period of time. Let $X_{t_{1},j}^{t_{2}}=\{{\hat{\text{EMES}}}_{t_{1}+1:t_{1}+h}^{j},\ldots ,{\hat{\text{EMES}}}_{t_{2}+1:t_{2}+h}^{j}\}$, where $t_{1}<t_{2}$ and j=1,…,N. Similar to [42], the similarity matrix during period *t*_1_ to *t*_2_ is composed of the cosine similarity between pair of firms *i* and *j*:

$$\rho_{t_{1},ij}^{t_{2}}=\frac{X_{t_{1},i}^{t_{2}⊤}X_{t_{1},j}^{t_{2}}}{\left\|X_{t_{1},i}^{t_{2}}\right\|\left\|X_{t_{1},j}^{t_{2}}\right\|}\;\text{for}i,j=1,\ldots ,N,\text{and}j\neq i.$$ (1)

For the purpose of interpretation, the asymmetric breakpoint approach is employed to partition entries in the similarity matrix into three group: strong positive, strong negative and zero correlation. Specifically, let $\rho_{t_{1}}^{t_{2}}=\left(\rho_{t_{1},1}^{t_{2}},\rho_{t_{1},2}^{t_{2}},\ldots ,\rho_{t_{1},n}^{t_{2}}\right)⊤$ to be the vector of similarities with $\rho_{t_{1},1}^{t_{2}}<\cdots <\rho_{t_{1},n}^{t_{2}}$, where n=N(N−1)/2. We further divide $\rho_{t_{1}}^{t_{2}}$ into the positive vector $\rho_{t_{1}}^{t_{2},+}=\left(\rho_{t_{1},1}^{t_{2},+},\ldots ,\rho_{t_{1},n_{1}}^{t_{2},+}\right)⊤$ and the negative vector $\rho_{t_{1}}^{t_{2},-}=\left(\rho_{t_{1},1}^{t_{2},-},\ldots ,\rho_{t_{1},n_{2}}^{t_{2},-}\right)⊤$, $n_{1}+n_{2}=n$. We estimate the break fractions ${\hat{\theta }}^{+},{\hat{\theta }}^{-}$ using the method proposed by [75], and construct the adjacency matrix $A_{t_{1}}^{t_{2}}$, whose (*i*,*j*) entry is given by

$$a_{t_{1},ij}^{t_{2}}=\left\{ \begin{array}{cc} 1 & \text{ if }\rho_{t_{1},v^{+}(i,j)}^{t_{2},+}>\rho_{t_{1},{\hat{\theta }}^{+}}^{t_{2},+}\\ -1 & \text{if }\rho_{t_{1},v^{-}(i,j)}^{t_{2},-}<\rho_{t_{1},{\hat{\theta }}^{-}}^{t_{2},-}\\ 0 & \text{ otherwise } \end{array}\right.$$ (2)

where $v^{+}(i,j)$ assigns the index of $\rho_{t_{1}}^{t_{2},+}$ to the pair (*i*,*j*), and ${\text{v}}^{-}(\text{i},\text{j})$ assigns the index of $\rho_{t_{1}}^{t_{2},-}$ to the pair (*i*,*j*). $\rho_{t_{1},{\hat{\theta }}^{+}}^{t_{2},+}$ and $\rho_{t_{1},{\hat{\theta }}^{-}}^{t_{2},-}$ are the breaking points calculated based on ${\hat{\theta }}^{+},{\hat{\theta }}^{-}$.

To quantify the aggregate risk, we calculate the total systemic risk score $S_{t_{1}}^{t_{2}}$ as follow


$$S_{t_{1}}^{t_{2}}({\bar{C}}_{t_{1}}^{t_{2}},A_{t_{1}}^{t_{2}})={\bar{C}}_{t_{1}}^{t_{2}⊤}A_{t_{1}}^{t_{2}}{\bar{C}}_{t_{1}}^{t_{2}}$$

where ${\bar{C}}_{t_{1}}^{t_{2}}=\left({\bar{C}}_{t_{1},1}^{t_{2}},\ldots ,{\bar{C}}_{t_{1},N}^{t_{2}}\right)⊤\in {\mathbb{R}}^{N}$ is the vector of nodal averaging market capitalizations during period *t*_1_ to *t*_2_. Therefore, for firm *j*, the systemic risk score is given by

$$S_{t_{1},j}^{t_{2}}=\frac{\partial S_{t_{1}}^{t_{2}}}{\partial {\bar{C}}_{t_{1},j}^{t_{2}}}\cdot {\bar{C}}_{t_{1},j}^{t_{2}}=\left(2\sum_{i=1}^{N}a_{t_{1},ji}^{t_{2}}{\bar{C}}_{t_{1},i}^{t_{2}}\right){\bar{C}}_{t_{1},j}^{t_{2}}.$$ (3)

### 3.3 Dynamic network quantile regression model

To further investigate how other factors affect the extreme risk of energy companies under large shocks, we adopt the DNQR model proposed by [43]. This model enables us to analyze the impact of firm-specific characteristics and market environment after controlling for the effects of other energy companies with risk contagion in both the current and lagged periods. In the DNQR model, the α-th conditional quantile of firm *j*’s return $R_{t}^{j}$ is modeled as

$$Q_{R_{t}^{j}}\left(\alpha |F_{t}\right)=\gamma_{0}^{0}(\alpha )+\sum_{l=1}^{q_{1}}\theta_{l}^{0}(\alpha )Z_{jl}+\gamma_{1}^{0}(\alpha )\sum_{i=1}^{N}w_{ji}R_{t}^{i}\;\;+\gamma_{2}^{0}(\alpha )\sum_{i=1}^{N}w_{ji}R_{t-1}^{i}+\gamma_{3}^{0}(\alpha )R_{t-1}^{j}+\sum_{k=0}^{q_{2}}F_{t-k}^{⊤}\beta_{k}^{0}(\alpha )$$ (4)

where $F_{t}$ denotes the information available at time *t*, *Z* represents the firm specific characteristics, *F* represents other common macroeconomic and financial factors. The weight matrix *w*_*ji*_ is constructed based on (2), where *w*_*ji*_ takes 0 when *a*_*ji*_ takes values of 0 and –1, indicating no or negative correlation between company *j* and company *i*. When *a*_*ji*_ takes the value of 1, indicating a positive correlation between company *j* and company *i* that could potentially lead to systemic risk contagion, *w*_*ji*_ is set to 1. The first component, $\gamma_{0}^{0}(\alpha )+\sum_{l=1}^{q_{1}}\theta_{l}^{0}(\alpha )Z_{jl}$ measures the quantile-specific nodal effects of characteristics for firm *j*, aiming to control for the influence of the firm’s intrinsic attributes on its extreme risk. Network spatial correlations among nodes are captured by $\sum_{i=1}^{N}w_{ji}R_{t}^{i}$, while network temporal correlations among nodes are captured via $\sum_{i=1}^{N}w_{ji}R_{t-1}^{i}$. Hence, $\gamma_{1}^{0}(\alpha )$ captures the quantile-specific contemporary network effects, reflecting how the current returns of positively connected firms influence the extreme returns of firm *j* at time *t*, which helps identify the immediate spillover effects among energy companies during market shocks. In contrast, $\gamma_{2}^{0}(\alpha )$ captures the lagged diffusion network effects. The quantile-specific momentum effect is measured via $\gamma_{3}^{0}(\alpha )$, indicating how a firm’s past returns influence its current extreme returns. In the last term, the impact of common macroeconomic and financial factors is considered, allowing $\beta_{k}^{0}(\alpha )$ to capture the influence of these external shocks on a firm’s extreme returns.

## 4 Empirical results and discussion

### 4.1 Data and descriptive statistics

In this paper, we examine the risk profiles of the global energy market, as represented by the S&P Global Platts Top 250 Global Energy Company Rankings of 2021 which could be found at *https://www.spglobal.com/commodityinsights/top250/rankings/2021* in the face of large shocks. As found by [2, 4], due to the dominant position of top energy companies in the global energy system, studying the level of interconnectedness among them is crucial for understanding the systemic risks in the global energy market. Based on their research, we expand our selection of companies to all 250 companies, hoping to provide a more comprehensive examination of the risk-generating mechanisms in the global energy markets. After filtering out companies with missing data, we obtain a sample of 192 energy companies from 34 countries across three regions, Asia/Pacific, EMEA and Americas. For the top energy companies from the Chinese mainland market, we exclude companies that have been suspended for more than three months in order to ensure the reliability of the calculation of the systemic risk indicator. They belong to 9 industries including Coal and Consumable Fuels (CCF), Electric Utilities (EU), Multi- Utilities (MU), Gas Utilities (GU), Independent Power Producers and Energy Traders (IPPET), Integrated Oil and Gas (IOG), Oil and Gas Storage and Transportation (OGST), Oil and Gas Exploration and Production (OGEP), Oil and Gas Refining and Marketing (OGRM). Firm-level returns are calculated using the natural logarithmic differences of daily closing prices. In order to cover a wider range of energy companies, the sample interval is from January 1, 2007 to August 31, 2022, with a total of 3944 observations. In measuring the systemic risk of the top energy companies, we use a 1-year rolling window, so that the actual systemic risk indicator calculated starts from January 2008. According to [1, 19], during this sample period, five significant shocks are encompassed: Global financial crisis (January 2008 to May 2009), European debt crisis (October 2009 to December 2012), Brexit Referendum (From January to December 2016), US–China trade disputes (August 2017 to July 2018), and COVID-19 epidemic (January 2020 to August 2022). The remaining periods are considered as normal periods.

**Fig 1:**
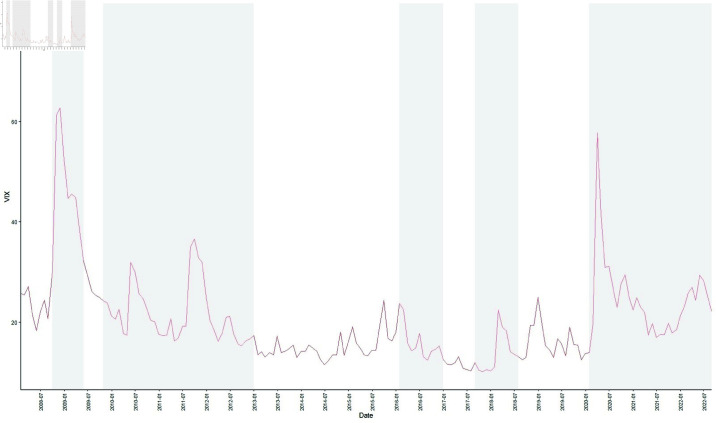
Time series of VIX index among top energy companies from January 2008 to August 2022. The gray areas from left to right represent extreme events that may have an impact on the energy market. The gray shaded areas from left to right represent the periods of the global financial crisis, the European debt crisis, the Brexit Referendum, the US–China trade disputes, and the COVID-19 pandemic.

The selection of these events is primarily based on the following considerations: First, these events induced substantial market volatility at the global level. The time series of the VIX index, Fig 1 commonly referred to as the market panic index, illustrates the volatility during these major shocks, with the shaded grey regions highlighting the five selected events. It is evident that the VIX index exhibited sharp movements during these periods, indicating the profound impact these events had on market stability. Second, these events encompass different types of shocks, including financial crises (the global financial crisis and European debt crisis), political events (the Brexit referendum and the US–China trade dispute), and public health crises (the COVID-19 pandemic). This variety allows for a comprehensive assessment of the diverse impacts of external shocks on the energy market. Finally, as noted in the literature, these events had substantial practical effects on the energy market. For example, both the global financial crisis and the European debt crisis directly affected the volatility of commodity prices [2, 76]. The US–China trade dispute and the COVID-19 pandemic, on the other hand, altered the global supply-demand dynamics of energy products [19, 77].

Basic information on our selected sample of energy companies is presented in S1_appendix, including the companies’ key financial metrics provided by S&P Global and descriptive statistics of their stock returns. Geographically, 72 of these companies are from Asia and the Pacific, 52 from Europe and the Middle East, and 68 from the Americas, with the largest number of companies from the US at 50, followed by China with 34 companies on the list, and then Japan with 14. In terms of industry distribution, there are approximately 60 electric power and oil-related energy companies, which represents an absolute advantage compared to other industries from a quantitative point of view. This indicates that traditional energy-related companies still hold a dominant position in the global energy market. In addition, all of the top global energy companies in the coal-related industries are from China, highlighting the significant market position of coal in China’s energy consumption. This phenomenon also implies that China faces more serious challenges in the process of green and low-carbon energy transition.

Descriptive statistics of financial indicators and stock returns of energy companies show that companies with higher asset values tend to have substantial revenues but may not necessarily earn corresponding profits and stock returns. For example, Shell and Exxon rank second and fourth in asset value and third and fifth in revenue, but their profits are negative. Gazprom, a company from Russia, ranks in the top 10 in terms of assets and revenue and generates positive profits, yet its average stock return ranks eighth from the bottom. On the other hand, PetroChina ranks in the top three globally for both assets and revenue, generates positive profits, and has a higher average stock return, indicating that the company possesses a higher quality within the global energy market.

When measuring the systemic risk of energy companies, we employ a multi-layer hierarchical dependence structure in the form of “US (sys)-Country/Region-Firms”. For the US, we select the S&P 500 Index as the market index, which is also used to define systemic events. The descriptive statistics of the market indices of each country/region are displayed in Table 1. As can be seen from the table, the average return of India’s Nifty 50 Index reached the highest at 0.0293% during the sample period, followed by the OBX Index of Norway and the S&P 500 Index of the US with 0.0282% and 0.0260%. Importantly, all three countries mentioned are significant producers of oil and natural gas, indicating the rationale behind employing the hierarchical structure to analyze the systemic risk of energy companies.

**Table 1:** This table presents the descriptive statistics of market indices’ daily returns in the countries where the top energy companies are located. The mean (Mean, in percentage), standard deviation (Std.), skewness (Skew.), kurtosis (Kurt.) are shown from January 1, 2007 to August 31, 2022.

Country/Region	Market stock index	Mean	Std.	Skew.	Kurt.
Australia	S&P/ASX 200	0.005	0.011	–0.687	7.398
China	CSI 300 Index	0.018	0.017	–0.554	3.945
Hong Kong, China	Hang Seng Index	–0.001	0.015	0.021	7.947
Taiwan, China	TSEC Weighted Index	0.017	0.012	–0.464	4.318
India	Nifty 50 Index	0.029	0.011	–0.224	19.666
Japan	Nikkei 225 Index	0.013	0.015	–0.448	7.711
South Korea	KOSPI Composite Index	0.014	0.012	–0.509	9.467
Malaysia	FTSE Bursa Malaysia KLCI	0.008	0.008	–0.836	12.605
Philippines	PSEi Index	0.021	0.013	–1.027	11.196
Singapore	STI Index	0.002	0.011	–0.263	7.423
Thailand	SET Index	0.024	0.012	–0.943	11.479
Austria	Austrian Traded Index	–0.012	0.016	–0.478	7.868
Belgium	BEL 20 Index	–0.005	0.013	–0.616	10.390
Czech Republic	PX Index	–0.009	0.013	–0.662	17.542
Finland	OMX Helsinki 25 Index	0.012	0.014	–0.221	4.616
France	CAC 40 Index	0.002	0.014	–0.256	7.882
Germany	DAX Performance Index	0.016	0.014	–0.195	7.856
Greece	ASE Composite Index	–0.043	0.020	–0.467	6.880
Hungary	Budapest Stock Exchange Index	0.013	0.015	–0.431	9.192
Italy	FTSE MIB Index	–0.017	0.016	–0.643	9.082
Netherlands	AEX Index	0.008	0.013	–0.364	9.002
Norway	OBX Index	0.028	0.015	–0.601	7.638
Poland	WIG20 Index	–0.020	0.015	–0.507	6.079
Portugal	Portuguese Stock Index	–0.016	0.013	–0.379	6.774
Qatar	Qatar Exchange Index	0.016	0.012	–0.632	11.767
Russia	MOEX Russia Index	–0.007	0.019	–3.298	96.162
Saudi Arabia	Tadawul All Share Index	0.012	0.014	–1.267	12.992
Spain	IBEX 35 Index	–0.010	0.013	–0.388	40.627
Sweden	OMX Stockholm 30 Index	0.013	0.014	–0.178	5.523
Switzerland	Swiss Market Index	0.005	0.011	–0.394	9.349
United Arab	DFM General Index	–0.005	0.016	–0.168	7.934
UK	FTSE100 Index	0.003	0.012	–0.386	9.584
Brazil	IBOVESPA Index	0.023	0.018	–0.446	9.547
Canada	S&P/TSX Composite Index	0.010	0.012	–1.047	19.709
Chile	S&P/CLX IPSA Index	0.018	0.012	–0.772	18.904
US	S&P 500 Index	0.026	0.013	–0.547	12.230

Following [54], we define systemic events based on the dynamics of VaR. The macro-economic variables we employ to predict VaR are listed in Table 2. Additionally, with reference to [78] and [4], we also examine the impact of external factors such as total assets (Size), operating margin (OM), and debt/equity (DE), as well as the economic policy uncertainty (EPU), WTI crude oil price (WTI), the crude oil volatility index (OVX), the Bloomberg Barclays Global Aggregate Index (BBGA), the US Dollar price index (DXY), and the S&P 500 implied volatility index (VIX) on the tail risk of energy companies. The historical Treasury bill rates and Moody’s Baa bond returns used in our study are downloaded from the Federal Reserve Bank of St. Louis website. The long-term bond yield data are obtained from the US Department of the Treasury website. The economic policy uncertainty (EPU) and the monetary policy uncertainty (MPU) data are sourced from [79]. The real estate sector return, financial sector return, and 3-month LIBOR rate are obtained from Bloomberg. All the stock market indices, energy company stock prices and their financial data, as well as other data used in this paper, are obtained from Thomson Reuters Datastream.

**Table 2:** Descriptions of macroeconomic, firm financial and market environment variables.

Type	Variables	Description
Macroeconomic	Market volatility	22-day rolling standard deviation of the market return
	Real estate excess return	Real estate sector return minus financial sector return
	Credit spread change	Moody’s Baa bond return minus ten-year Treasury bill rate
	Short-term TED spread	Three-month LIBOR rate minus three-month Treasury bill rate
	Term spread change	Long-term bond yield minus three-month Treasury bill rate
	3-month yield change	Change in the three-month Treasury bill rate
Firm financial	Size	Total assets
	Debt	Debt/Equity
	Profitability	Operating Margin
Market environment	VIX	S&P 500 implied volatility index
	OVX	Crude oil volatility index
	WTI	WTI crude oil price
	DXY	the US dollar price index
	EPU	Economic policy uncertainty
	MPU	Monetary policy uncertainty

### 4.2 Internal perspective: the risk formulation mechanism of the global energy market

In this section, we first decompose the systemic risk of top energy companies from both a temporal and a spatial perspective. Subsequently, we analyze the risk correlation among energy companies under large shocks, thereby examining the internal risk generation mechanisms of the global energy market.

#### 4.2.1 Internal perspective from the time dimension: systemic risk decomposition of top energy companies.

**Fig 2:**
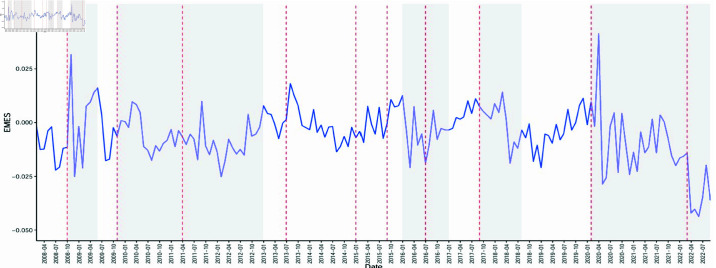
Time series of average systemic risk among top energy companies from January 2008 to August 2022. The red dashed lines from left to right represent extreme events that may have an impact on the energy market. The gray shaded areas from left to right represent the periods of the global financial crisis, the European debt crisis, the Brexit Referendum, the US–China trade disputes, and the COVID-19 pandemic.

Fig 2 presents the time series of average systemic risk measured by EMES of top global energy companies from January 2008 to August 2022. For ease of recognition, we have marked the extreme events that could have an impact on the energy market with red dotted lines. From left to right, these events include the bankruptcy filing of Lehman Brothers in September 2008, the Greek government’s announcement of fiscal deficit and public debt exceeding EU limits in October 2009, the massive tsunami triggered by the Great East Japan Earthquake in March 2011, leading to a significant release of radioactive materials from the Fukushima nuclear power plant, the global financial market turbulence caused by the former Federal Reserve Chair Ben Bernanke’s announcement in June 2013 of a potential tapering of quantitative easing in 2014, the lack of consensus on production cuts by OPEC in December 2014, resulting in a sharp decline in international oil prices, the global stock market crash in August 2015, the Brexit referendum in June 2016, the escalation of US–China trade tensions following President Trump’s signing of executive memoranda in August 2017, the outbreak of the COVID-19 pandemic in January 2020, and the outbreak of the Russia–Ukraine conflict in February 2022.

Overall, the EMES metrics we use are basically accurate in capturing the impact of extreme events on the systemic risk in the energy market. For example, EMES shows varying degrees of increase shortly after the Fukushima nuclear disaster in March 2011 and the failure of OPEC to agree on production cuts in December 2014. In particular, it experiences a sharp rise after the bankruptcy of Lehman Brothers and the Brexit Referendum and outbreak of the COVID-19 pandemic, reflecting the enormous impact of these “black swan” events on the global energy market. Meanwhile, our market-information-based systemic risk measure allows us to observe the impact of dramatic shocks in financial markets on the stock returns of top energy companies. For example, in addition to the global financial crisis, we also capture the impact of the global stock market crash in August 2015. Since the decline in stock prices may lead to a shrinkage in a company’s market capitalization and thus affect the financing activities of listed companies, this non-decentralized market systemic risk on energy companies cannot be ignored. Besides, our metrics also incorporate the influence of macroeconomic and regional market environment, which enables us to capture the impact of regional risk events such as the Fed’s monetary policy changes. Based on the results manifested by this time series plot, we find that the systemic risk level of global top energy companies exhibits significant volatility, especially after extreme risk events. This indicates that studying the systemic risk of top energy companies is of great importance in providing guidance for stabilizing the international energy market, making it sustainable and healthy.

The gray shaded areas in Fig 2 highlight the periods during the global financial crisis, the European debt crisis, the Brexit Referendum, the Sino-US trade conflict and the COVID-19 pandemic. Compared to normal times, the patterns of systemic risk under these great shocks are similar, with all experiencing more intense fluctuations. During the initial outbreak of the global financial crisis and the COVID-19 pandemic, the global equity market was significantly affected and experienced a sharp decline. These crisis events led to a rapid increase in systemic risk and therefore brought unprecedented shocks to the stability of the energy market. During the European debt crisis, the Brexit Referendum, and the US–China trade conflict, the average EMES of top energy companies, although not as volatile as during the former two great shocks, still experienced increases to some extent. It is worth noting that the increase of systemic risk is somewhat lagged from the occurrence of each shock event. This reminds us to track the long-term sustained effects of these events while focusing on their initial impacts.

**Fig 3:**
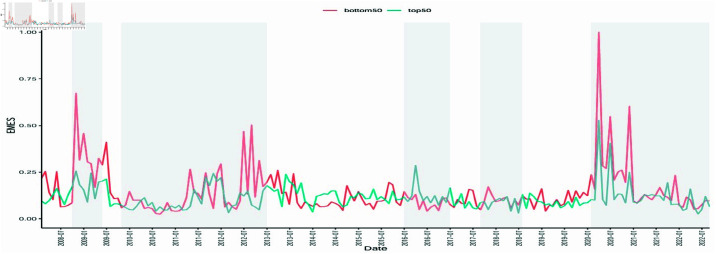
Time series of maximums for the systemic risk of the top 50 and bottom 50 companies in the Platts ranking. The gray shaded areas from left to right represent the periods of the global financial crisis, the European debt crisis, the Brexit Referendum, the US–China trade disputes, and the COVID-19 pandemic.

Fig 3 shows the two time series of maximum systemic risk for the top 50 (blue solid line) and the bottom 50 (red solid line) ranked energy companies according to Platts, respectively. This reflects the maximum systemic risk for companies in the upper 20% and the lower 20% percentiles among the top 250 global energy companies. We find that both groups exhibit similar patterns under the impact of large shocks. However, the maximum systemic risk level for the bottom 50 companies is significantly higher than that for the top 50 companies. For example, at the early stage of the global financial crisis, Pioneer Natural Resources, ranked 227th, had a highest EMES level of 0.67. Similarly, during the global spread of the COVID-19 pandemic, Plains All American Pipeline, ranked 226th, experienced a significant drop in stock price from $19.27 on January 6, 2020 to $3.29 on March 18, 2020, representing a decline of over 80%. And its EMES was close to 1, which captured the significant systemic risk the company faces. Since S&P Global ranks energy companies based on four main financial metrics: asset worth, revenues, profits and return on invested capital. This also reflects that in addition to relying on the market performance of energy companies to analyze their risk volatility, it is necessary to consider the impact of the company’s financial condition on its systemic risk.The only exception was during the 2016 Brexit referendum, when the overall systemic risk level of top ranked companies was higher than that of bottom ranked companies. This is because the direct impact of Brexit on regions outside of Europe is relatively small, and 12 out of the top 50 energy companies are from Europe, whereas only 5 out of the bottom 50 energy companies are from Europe. At the same time, there are a large number of Chinese energy companies among the top energy companies, with 3 out of the top 10 and 6 out of the top 50 from China. In 2016, In 2016, China began to promote “supply side reform” and eliminate excess capacity. Under this impact, top-ranked Chinese energy companies contribute more risk in the top50 sample due to domestic factors. This can be seen from the subsequent scatterplot of risk distribution, where the risk level of Chinese energy companies during the Brexit period is significantly higher than that during the European debt crisis. This indicates that although the market performance of the top-ranked top energy companies is better than that of the bottom-ranked companies in the case of major shocks, they are still likely to be exposed to higher risks in the case of direct local shocks.

**Fig 4:**
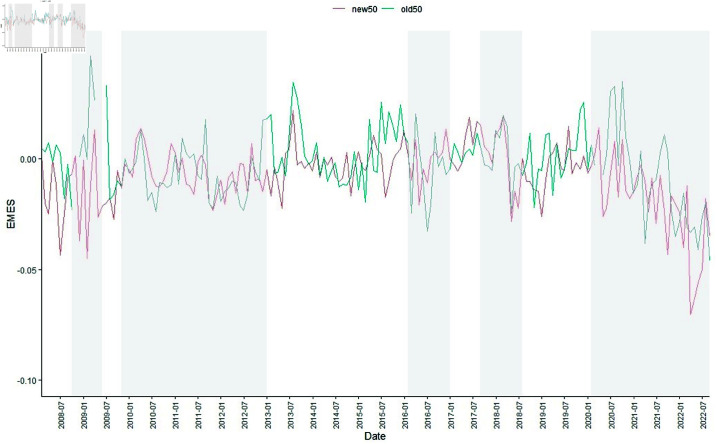
Time series of maximums for the systemic risk of the earliest 50 and latest 50 companies in the Platts ranking. The gray shaded areas from left to right represent the periods of the global financial crisis, the European debt crisis, the Brexit Referendum, the US–China trade disputes, and the COVID-19 pandemic.

We plotted the EMES time series of the 50 energy companies with the earliest establishment time and the 50 energy companies with the latest establishment time in the total sample as sub samples. As shown in the Fig 4, their trends are generally consistent over time, but the difference is that the risk level of newly established companies is lower than that of earlier established companies. As shown in the figure, their trends are generally consistent over time, with the difference being that the risk level of the sample of newly established companies is lower than that of the earlier established companies. There are several reasons for this result. Firstly, newly established energy companies generally have smaller asset sizes compared to the earlier established companies, lower levels of internationalization in their business activities, and are relatively less affected by the international energy market. Secondly, 30 of the 50 newest energy companies are from developing countries, which tend to have the backing of state power and monopolistic nature, reducing the risk in the face of major shocks. On the contrary, 46 of the 50 earliest energy companies are from developed countries in Europe and the United States, and 27 of them are from the United States. These early established energy companies operate globally in a market-based manner, have accumulated huge assets and complex energy networks, making them more exposed to major shocks. The general consistency in risk trends between the two samples of firms reflects the integration of the global energy market, suggesting that we could take a network approach to studying risk contagion in energy markets.

#### 4.2.2 Internal perspective from the spatial dimension: systemic risk decomposition of top energy companies.

**Fig 5:**
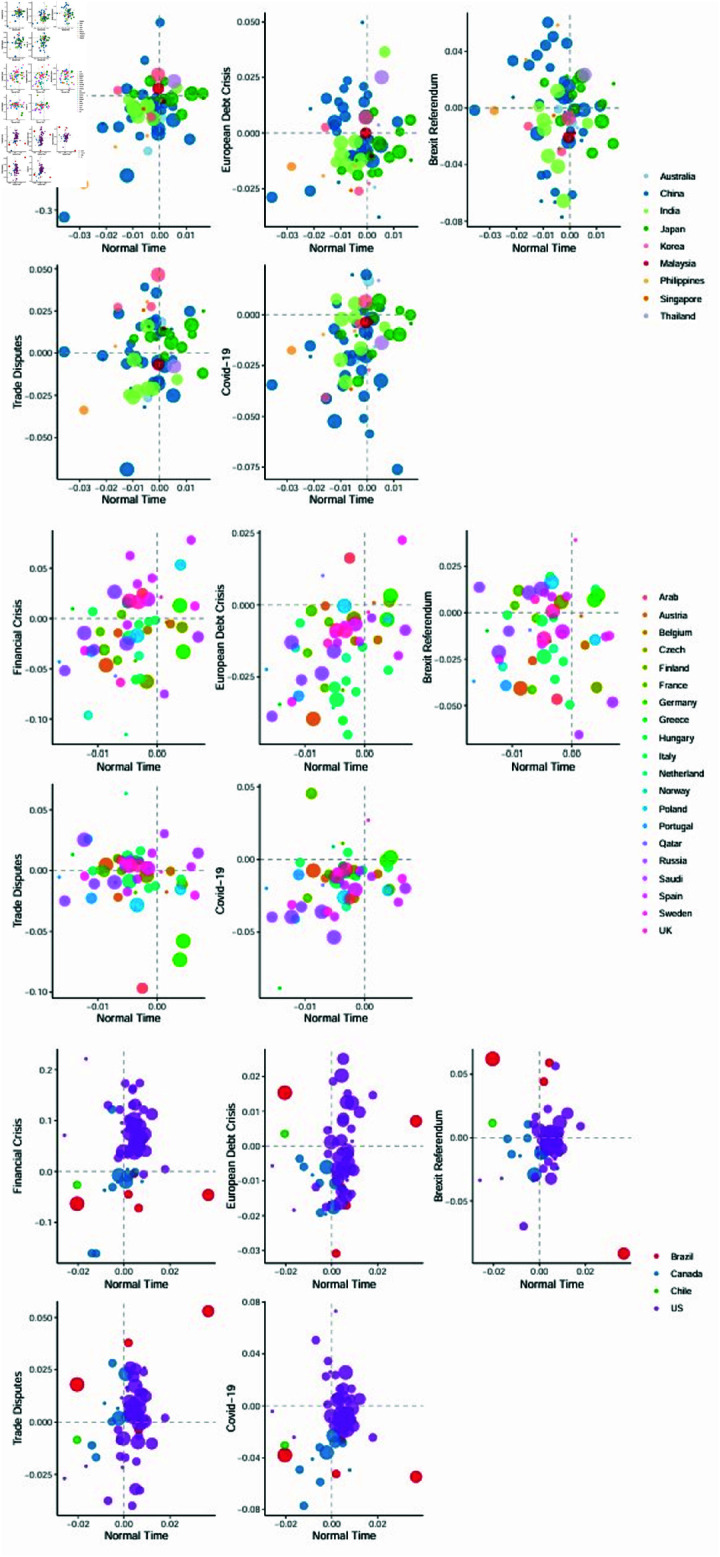
Scatter plots of the systemic risks of energy companies located in Asia/Pacific, EMEA, and Americas during periods of large shocks and normal times. The horizontal axis represents the systemic risk during normal periods, while the vertical axis represents the systemic risk levels in the period of large shocks. Different colors are used to differentiate the countries to which the energy companies belong, and the size of the circles corresponds to the companies’ positions in the Platts Rankings, with larger circles indicating higher rankings (top 50, 51–100, 101–200, and 201–250).

To further examine the geographic peculiarities of the top energy companies’ systemic risks across time, we provide in Fig 5 “normal vs. crisis” periods scatter plots of the systemic risks for energy companies based in Asia/Pacific, EMEA, and Americas. The horizontal axis in these figures reflects systemic risks during normal periods other than the four significant shocks, while the vertical axis represents systemic risks during the five large shocks. The countries to which the energy businesses belong are differentiated by different colors, and the size of the circles correlates to the companies’ positions in the Platts Rankings, with larger circles signifying higher rankings (top 50, 51–100, 101–200, and 201–250). The scatter plots are divided into four parts by two dashed lines based on the value of zero. The upper-left parts indicate that the energy companies have a negative EMES during normal periods but positive EMES during the corresponding large shock periods, and the same for the other parts.

Compared to normal periods, the global financial crisis has the strongest impact on the systemic risks of top global energy companies, followed by the effects of the COVID-19 pandemic and the US–China trade disputes, and the European debt crisis has the lowest impact on the systemic risks of energy companies. This is caused by the different impacts of several large shocks on the global economy.The Global Financial Crisis (GFC) first erupted in the United States, which was the center of the global economy at that time, and then quickly spread to the world and triggered a global recession. While the European debt crisis broke out in Greece, and then spread to Spain, Portugal, Ireland and Italy, which brought about an overall recession in the eurozone, but the impact outside the eurozone was relatively small. The sources of large shocks and their overall impact on the economy and society have a significant impact on the systemic risk of energy companies and energy market. In Fig 5, the absolute value of the EMES during the global financial crisis is the highest among the five shock events, while in other periods, although some energy companies have positive EMES, the values are relatively small. In addition, the systemic risks of top-ranking energy companies in the Platts rankings are generally more stable. In most cases, the top 50 energy companies are less affected by extreme shocks, while the lower-ranked companies are more prone to sharp increases in systemic risks during large shocks. This suggests that the systemic risks of energy companies are not only affected by common risk factors under large shocks, but also by their individual characteristics.

Meanwhile, systemic risks among global energy companies during large shocks exhibit clear clustering phenomena. From a regional perspective, energy companies, whether from Asia/Pacific, EMEA, or the Americas, are for the most part concentrated on one side of the dotted line or at the junction of the two dotted lines. For example, during the global financial crisis, a majority of energy companies from the Asia/Pacific region cluster around the intersection of the two dashed lines, while European energy companies are primarily located to the left of the dashed line, and energy companies from the Americas are predominantly situated above the dashed line. During the COVID-19 pandemic, most energy companies from the Asia/Pacific and Europe are concentrated below the dashed line, while energy companies from the Americas are clustered around the intersection of the dashed lines.

At the country level, the risk clustering phenomena are most prominent among energy companies from the Americas. This is primarily due to the fact that 50 out of the top energy companies in the Americas region are from the United States, accounting for a high percentage of 73.5%. These energy companies are exposed to similar external risk shocks during large shocks periods and therefore exhibit similar systemic risk performance. A typical example is that during the global financial crisis, the systemic risks of almost all top US energy companies are greater than 0, indicating that the U.S. energy market faces significant systemic risk threats at this time. Given the strong linkage between financial and energy markets [80], we believe that the severe collapse of the US stock market has resulted in a significant devaluation of energy companies’ market capitalization. At the same time, the financial crisis has led to a sluggish real economy, causing a decline in energy demand, which also poses a threat to the security of U.S. energy companies. These factors are likely to result in energy companies facing a crisis due to a lack of liquidity.

Compared to the Americas, there are numerous countries in Europe and the Middle East. Despite the dispersed distribution of energy companies across these countries, they also exhibit regional-level clustering characteristics. For example, in normal times, most energy companies in European countries have lower systemic risks, except for two major Germany utility companies E.ON and RWE. These two German companies are ranked 28th and 46th, and they have consistently faced higher risk levels due to traditional business burdens in the context of Germany’s energy transition. Another example is that within the EMEA region, many of the energy companies listed in the Platts ranking originate from Italy, Spain, and Russia. Therefore, during the periods of the global financial crisis and the European debt crisis, energy companies from these three countries exhibit a noticeable consistency in their systemic risk performance. In Asia, during periods of large shocks, systemic risks of energy companies in China, Japan, and South Korea often r cluster together around the origin. In contrast, those in India remain relatively isolated. This phenomenon could be attributed to the economic connections among China, Japan, and South Korea in East Asia. Meanwhile, many of the Indian energy companies on the list are highly-ranked oil companies with monopolistic market positions and stable business operations within their country, making them less susceptible to the impact of shocks.

Furthermore, with the exception of the direct impact of domestic supply-side reforms with the same period during Brexit Referendum, Chinese energy companies have maintained relatively low levels of systemic risk overall under large shocks. This is mainly because most of the Chinese energy enterprises selected for the Platts ranking are state-owned enterprises with large scale, good financial conditions, and high market positions. They primarily provide essential energy resources such as coal, electricity, natural gas, and oil required for production and daily life, thus possessing strong risk resilience capabilities. However, considering that China’s energy security may still face severe external challenges, we further analyze the systemic risk characteristics of Chinese energy companies during large shocks. We find that the systemic risks of Chinese energy companies are influenced by domestic and international market supply and demand. For example, during the global financial crisis, Shanxi Huayang Group New Energy Co., Ltd and Shanxi Coking Coal Energy Group Co., Ltd, both of which belong to the CCF industry, exhibited relatively high systemic risks. The reasons may stem from two aspects: On one hand, the significant impact of the financial crisis on the real economy led to a decrease in both domestic and international market demand, resulting in a continuous buildup of coal inventories and a substantial decline in coal prices. On the other hand, the price of oil dropped significantly from 147.25 per barrel on July 11, 2008, to 37.28 per barrel on January 14, 2009, which also had an impact on the coal market, causing it to become depressed. Another noteworthy fact is that during the US–China trade disputes, the imposition of tariffs by the US on China’s mechanical and electrical products amplified the impact of tariffs on the power equipment industry. In this situation, companies in the IPPET industry, such as Datang International Power Generation Co., Ltd, Huadian Power International Corp Ltd, and China Power International Development Ltd, faced higher threats of systemic risks. In addition, we also find that China’s macroeconomic regulation exerts an influence on the operation of energy companies. During the COVID-19 pandemic, the spread of the epidemic was rapidly controlled as the Chinese government quickly took proactive and effective measures to prevent the epidemic. At the same time, the Chinese government adopted a series of macroeconomic policies to promote economic recovery. Therefore, most energy companies in China were less exposed to risk during the COVID-19 pandemic and maintained a high degree of security.

**Fig 6:**
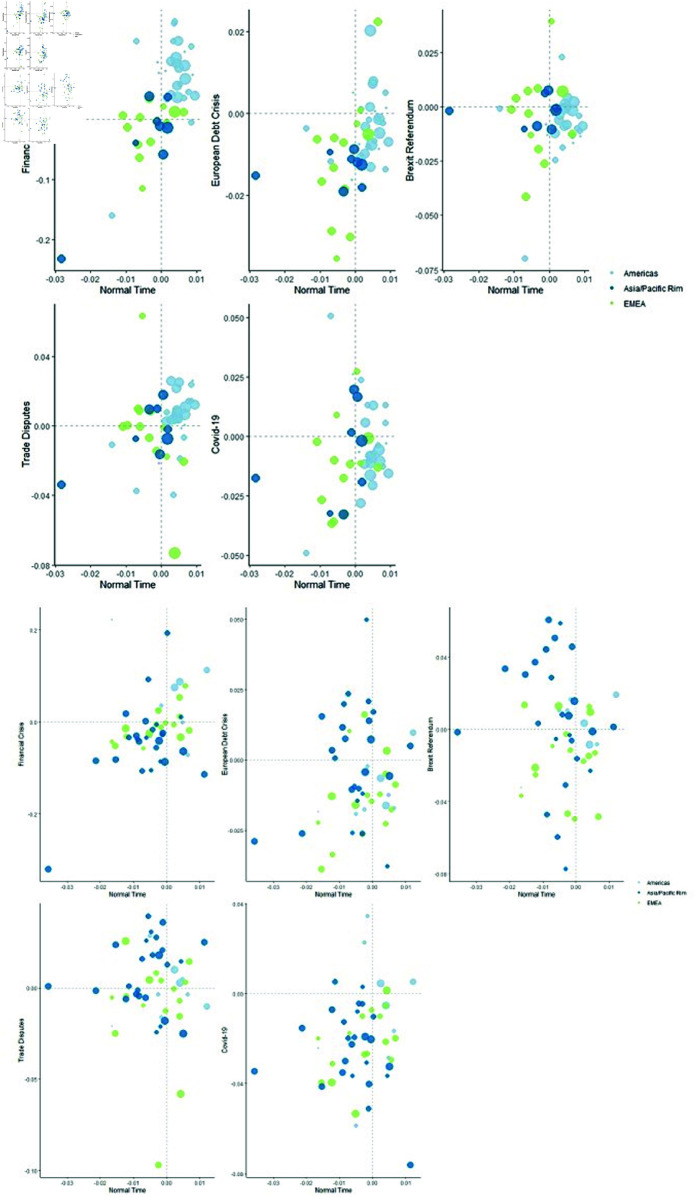
Scatter plots of the systemic risks of earliest 50 and latest 50 energy companies located in Asia/Pacific, EMEA, and Americas during periods of large shocks and normal times. The first set of images represents a sample of the earliest established companies, while the second set represents a sample of newly established companies. The horizontal axis and the vertical axis represents the same as Fig 5. Different colors are used to differentiate the regions to which the energy companies belong, and the size of the circles corresponds to the companies’ positions in the Platts Rankings, with larger circles indicating higher rankings (top 50, 51–100, 101–200, and 201–250).

After examining the risk profile of energy companies in the geospatial perspective, we then examined the risk distribution of the 50 earliest and latest established energy companies separately. As shown in the Fig 6, the first set images show the risk distribution of the earliest 50 established energy companies under major shocks, and the second set images show the risk distribution of the latest 50 established energy companies. One obvious feature of the two samples is the difference in the number of energy companies from the Americas and Asia Pacific regions, more precisely, the difference in the number of energy companies from the United States and China.In the first graph, a scatter plot reflecting the risk distribution of energy companies established earlier, more than half of the energy companies come from countries in the Americas, mainly the United States, while only few come from the Asia Pacific region. In the second graph, more than half of the energy companies come from the Asia Pacific region, mainly China. The number of energy companies from Europe and the Middle East has remained stable. This characteristic first reflects the close connection between energy and economy, with the United States, which has been the world’s largest economy since the early 20th century, occupying the most visible position in the energy sector. And China, today’s second largest economy, with the fastest-growing economy since the 21st century, has the most new top-tier energy companies.

The second significant feature is that the risk distribution of the two samples is completely different under major shocks, and this difference is determined by the source of major shocks. For example, when hit by the 2008 global financial crisis, which originated in the United States, the most America energy companies—mainly United States—were densely distributed in the upper right quadrant, while energy companies in the Asia Pacific, Middle East, and Europe were almost distributed in the lower half of the quadrant. In the first sample, American companies are predominant, while in the second sample, Chinese companies are predominant. Thus, the difference in risk distribution more reflects the different impact of this shock on the two economies.The second evidence comes from energy companies in Europe and the Middle East. In the two samples, the numbers are 13 and 16 respectively. The difference is that six of the new companies are from Russia and the Middle East, while all the old companies are from Western Europe or Northern Europe. It can be seen that there is no significant difference in the risk distribution of energy companies from this region under different shocks.Therefore, the research here shows that the source of major shocks and the corresponding geographical distribution of energy companies, rather than the timing of establishment, are more important for the risk performance of energy companies in different periods.

#### 4.2.3 Internal perspective: risk correlation structure of top energy companies under large shocks.

**Fig 7:**
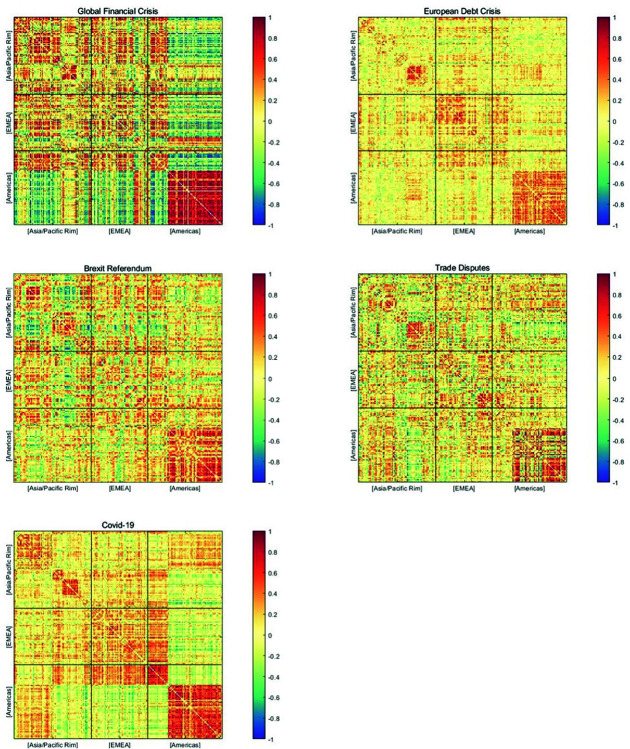
Similarity matrices during the global financial crisis, the European debt crisis, the Brexit Referendum, the US–China trade disputes, and the COVID-19 pandemic. The colors used to represent the degree of similarity vary from negative (blue) to positive (red) in the grids.

To further investigate the risk interactions among top energy companies under great shocks, we snapshot risk correlation networks around different significant events. These risk correlation networks are calculated using (1). Five network graphs illustrating the similarity of systemic risk profiles among energy companies during the global financial crisis, the European debt crisis, the Brexit Referendum, the US–China trade disputes, and the COVID-19 pandemic, are shown in Fig 7. Different regions are delineated by black lines, and the colors move from red to blue to signify positive to negative risk similarities (as shown in the colorbars).

According to these plots, top global energy companies demonstrate extensive risk correlations that exhibit dynamic change. Specifically, during the global financial crisis (the top left figure), compared to other periods, there was a higher intensity of risk interconnections among Asia/Pacific, Europe, and Canada and Brazil in the American regions, indicating the widespread impact of the financial crisis on the global energy market. For the United States, it is reasonable to observe a high level of risk similarity among its top energy companies, as the 2008 financial crisis led to a massive collapse in the US stock market, affecting even the leading energy companies listed therein. However, an interesting phenomenon is the remarkably negative risk similarity between the top energy companies in the United States and those in other regions. We conjecture that this may be attributed to a leading-lagging relationship in risk transmission between the United States and other countries during the financial crisis. In this paper, due to the availability of listed companies’ stock returns and the sample size limitations of our out-of-sample predictions when calculating EMES, we mainly focus on the impact of the global financial crisis triggered by the collapse of Lehman Brothers. However, the liquidity crisis in the United States preceding Lehman Brothers’ failure had already sent unfavorable signals to the financial market. After September 2008, the financial crisis began to spiral out of control, spreading to global financial markets and affecting globally listed energy companies. This is precisely the manifestation of the “cascading effect” of systemic risk.

During the European debt crisis (the top right figure), there was a relatively weak interaction on the systemic risks among the top global energy companies. However, regional risk aggregation still occurred within Europe and the United States. This may be attributed to the huge investment of US financial institutions in the Eurozone, thus leading the European debt crisis to pose a serious threat to US financial security. While during the Brexit Referendum period, the risk contagion network between regions was significantly weaker. Although Brexit brought a certain degree of uncertainty to the market, its consequences did not directly trigger an economic crisis, nor did it have a direct impact on the global energy supply chain. Therefore the risk contagion brought by the Brexit Referendum was also relatively small. In the period of the US–China trade disputes, there was a cross-regional risk comovement among top energy companies. Due to the close linkages in the global energy market, the deterioration of China-US energy trade relations led to the rapid transmission of risks to the global trading partners of both countries. In the case of the COVID-19 pandemic (the bottom right figure), the risk correlations among top energy companies exhibited more apparent regional effects. This is mainly because different countries’ governments implemented varying degrees of lockdown measures to prevent the spread of the epidemic, resulting in the accumulation of risks within the countries.

Additionally, a significant regional characteristic of systemic risks is demonstrated in the risk correlation matrices of the global top energy companies in times of large shocks. There are distinct red square areas within the Asia/Pacific, EMEA, as well as the Americas region, indicating the presence of widespread and stable risk comovement relationships within these regions. In the Asia-Pacific region, China, Japan, South Korea, and India all present significant intra-country risk similarities, with Japan and South Korea in particular showing a high degree of internal risk homogeneity. In Europe and the Middle East region, although energy companies are dispersed across numerous countries, the high level of economic integration within the European Union results in risk aggregation within individual countries as well as risk interactions among countries. In the Americas region, energy companies in Canada and the United States demonstrate dense and strong internal risk correlations, consistent with the findings of [2]. Our findings confirm that during great shocks, the risk interaction and accumulation within regions are stronger than cross-regional risk comovement.

Moreover, China, as the world’s second-largest economy and the largest energy consumer, exhibits unique risk characteristics within its energy market. Firstly, China’s leading energy companies have extensive global connections, resulting in significant risk comovements with top energy companies in Asia/Pacific, EMEA, and the Americas. Notably, the risk connections between Chinese and US energy companies have steadily intensified over time. Secondly, the risk contagion among Chinese energy companies is relatively weak, possibly due to industry monopolies and the prevalence of state-owned capital in China’s domestic energy sector. Hence, it can be inferred that the principal threats to China’s energy market security primarily stem from external sources rather than domestic ones. To ensure energy system security, China should bolster its forward-looking risk monitoring and response capabilities concerning imported risks, while also enhancing energy self-sufficiency and promoting energy transformation.

In the United States, during significant shock periods, its energy market demonstrates a strong risk comovement. Nevertheless, the strength of the risk linkage between US energy companies and those outside of the United States varies, but overall, they maintain a relatively independent position in the global energy risk network.

Meanwhile, we also examine the risk similarity matrices of several energy-exporting countries and regions, including the Middle East, Canada, Brazil, Norway, and Russia. As important participants in the global energy market, they not only exhibit risk homogeneity among themselves but also have significant risk comovement relationships with the external world. Besides, we discover that Canadian and Brazilian energy companies hold important positions in the risk interconnection network of the energy market. In previous studies, Canada and Brazil were often overlooked as minor players in the global energy market. Our research reveals that under the impact of the four major shocks, Canadian and Brazilian energy companies demonstrated significant risk comovements with energy companies in the Asia/Pacific and European regions, thus supplementing existing research.

The Russia–Ukraine conflict occurred on February 24, 2022, with huge ramifications for worldwide financial markets and the global energy circumstances. On the day the conflict broke out, Dow Jones futures and S&P 500 index futures both plummeted by over 2%, while Nasdaq 100 index futures experienced a sharp drop of approximately 3%. The VIX volatility index surged by over 12%. The Russian stock market suffered even more severe declines, with the US dollar-denominated RTS index falling by over 20%. In addition, during the month following the outbreak of the war, the global foreign exchange market experienced intense turbulence, with the US dollar index rapidly rising. As a result, the exchange rate of the Russian ruble against the US dollar depreciated by nearly half. Russia is one of the world’s most important energy-exporting countries, and Europe is its largest market for energy exports. The Russia–Ukraine conflict had a non-negligible impact on energy prices. On March 7, 2022, European natural gas futures prices approached $3,900 per thousand cubic meters. Brent crude oil briefly approached $140 per barrel, while WTI crude oil futures surpassed $130 per barrel, reaching new highs since 2008. Therefore, analyzing the impact of the Russia–Ukraine conflict on top energy companies is of great importance in understanding the mechanism of global energy market risk generation during large shocks.

**Fig 8:**
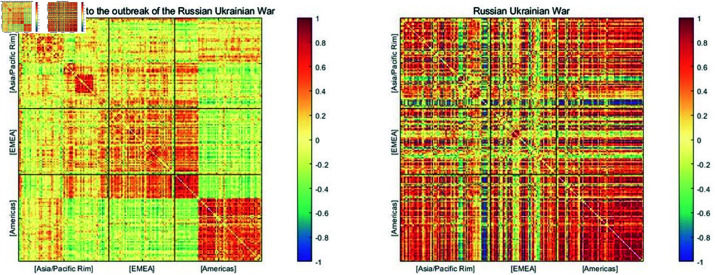
Similarity matrices from the outbreak of the COVID-19 pandemic to the eruption of the Russia–Ukraine conflict. The colors used to represent the degree of similarity vary from negative (blue) to positive (red) in the grids.

Fig 8 displays the risk similarity matrices among global top energy companies from the outbreak of the COVID-19 pandemic to the eruption of the Russia–Ukraine conflict and subsequently. It can be observed from the right plot in Fig 8 that the risk similarity matrix after the outbreak of the Russia–Ukraine conflict is covered by a large area of dark red, indicating strong positive risk similarities among world’s top energy companies. This suggests a significant level of risk interdependence and a high degree of risk comovement within the global energy system. The increase in energy prices caused by the Russian–Ukrainian conflict may raise production costs for some downstream energy corporations, in addition to changing the global energy trade network. While the adverse effects of the COVID-19 pandemic have not yet subsided, the impact of the Russia–Ukraine conflict will further threaten the stability of the global energy industry and highlight the high vulnerability of the global energy market during a period of overlapping crises. However, as the impact of the COVID-19 pandemic has not completely disappeared by the time of the Russo–Ukrainian conflict, and the sample period of the conflict included in our study is relatively short, overall, the impact of the COVID-19 pandemic still remains dominant. In future research, it is worthwhile to further investigate the mechanisms through which the Russo-Ukrainian conflict generates risks in the global energy market.

#### 4.2.4 Internal perspective: systemic risk scores of top energy companies under large shocks.

In the financial market, market capitalization can effectively reflect a company’s overall strength, encompassing factors such as profitability, industry status, and growth potential. Companies boasting larger market capitalization are generally more likely to secure sustained capital allocation. Conversely, they are also more susceptible to significant downturns in the event of a market crash during extreme circumstances. Consequently, in this section, we aim to amalgamate the risk interconnections among top energy companies with their respective market capitalization figures. Specifically, we will employ formula (2) to calculate the systemic risk score for each company. This approach allows us to holistically identify energy companies that occupy a substantial position within the global energy market risk network during large shocks.

Table 3. presents the top 20 systemic risk scores among top energy companies throughout the entire sample period. From a geographical perspective, these companies are widely distributed across Asia, Europe, and the Americas. Considering the countries from which these companies originate, they are primarily from China and the United States, reflecting the significant influence of the world’s two largest economies in the energy market. From the perspective of industry distribution, we find that among the top 20 global top-tier energy companies with the highest systemic risk scores during the sample period, the oil and gas industry dominates. A total of 16 companies belong to this industry, and all of the top 10 energy companies are integrated oil and gas companies. This reflects the crucial role played by oil and gas industry companies in risk contagion within the global energy market. After considering the interconnectivity between companies and market capitalization factors, PetroChina Co Ltd from China emerges as the most important energy company in the global energy market risk network. Additionally, we also find that Royal Dutch Shell plc, TotalEnergies SE, Chevron Corp, and Exxon Mobil Corp hold significant positions. This finding has also been confirmed in [2, 4].

**Table 3:** This table displays the top 20 systemic risk scores among top energy companies throughout the entire sample period.

Firm	Country	Industry	Systemic risk score
PetroChina Co Ltd	China	IOG	48362.220
Royal Dutch Shell plc	Netherlands	IOG	48188.317
TotalEnergies SE	France	IOG	40695.668
Public JSC Gazprom	Russia	IOG	28941.424
Eni S.p.A.	Italy	IOG	24446.674
Chevron Corp	US	IOG	23303.094
BP p.l.c.	United Kingdom	IOG	22281.608
Public JSC Rosneft Oil Co	Russia	IOG	16483.127
China Petroleum & Chemical Corp	China	IOG	15685.954
Repsol, SA	Spain	IOG	15530.735
CNOOC Ltd	China	OGEP	15396.790
Exxon Mobil Corp	US	IOG	15231.514
ENGIE SA	France	MU	12262.423
The Southern Co	US	EU	11532.811
Petroleo Brasileiro SA - Petrobras	Brazil	IOG	11142.150
Oil & Natural Gas Corp Ltd	India	IOG	10870.606
Reliance Industries Ltd	India	OGRM	10865.750
Duke Energy Corp	US	EU	8406.764
Equinor ASA	Norway	IOG	8361.721
NextEra Energy, Inc	US	EU	8353.721

We then examined the systematic risk score of newly established energy companies in the Table 4, which reflects both the market capitalization of the energy company itself and its risk connections with other energy companies. It can be seen that among the top 20 newly established energy companies, China Petroleum & Chemical Corp from the IOG industry is ranked the top 20 in terms of systematic risk score. In the total sample of 192 energy companies, 10 out of the 20 newly established energy companies ranked in the top 100 in terms of risk score, and 17 companies ranked in the top 150. We cannot ignore newly established energy companies and should continue to pay attention to their role in the energy market risk network. In addition, although the industry distribution of these newly established companies is relatively diversified, involving all 9 sub industries in the energy industry, 10 of these companies are directly related to the oil and gas industry. Combined with the industry distribution of the top 20 companies on systematic risk scores, it is clear that the oil and gas sector remains the most important and direct source of risk in the energy market at present.

**Table 4:** This table displays the systemic risk scores of 20 newly established top energy companies throughout the entire sample period.

Firm	Systemic risk score	Score_ranking	Country	Industry
China Resources Gas Group Ltd	431595.98	139	China	GU
PJSC Tatneft	3437791.596	49	Russia	OGEP
Abu Dhabi National Energy Co PJSC	1215807.296	96	United Arab	MU
China Shenhua Energy Co Ltd	5520245.924	37	China	CCF
China Power International Development Ltd	283606.056	153	China	IPPET
GS Holdings Corp	724263.0273	118	South Korea	OGRM
Qatar Gas Transport Co Ltd (Nakilat) (QPSC)	98031.90716	167	Qatar	OGST
China Coal Energy Co Ltd	984369.4814	103	China	CCF
China Gas Holdings Ltd	1871080.998	70	China	GS
Hera S.p.A.	563366.6811	129	Italy	MU
Energy Transfer LP	900560.0737	107	US	OGST
China Resources Power Holdings Co Ltd	1446531.177	83	China	IPPET
Shanxi Lu’an Environmental Energy Development Co, Ltd	402277.5442	141	China	CCF
Lundin Energy AB (publ)	2820958.96	54	Sweden	OGEP
Elia Group SA/NV	1114512.864	101	Belgium	EU
E.ON SE	4549170.016	41	Germany	MU
China Petroleum &Chemical Corp	15685953.86	9	China	IOG
ENN Energy Holdings Ltd	1384098.558	90	China	GU
Saudi Electricity Co	–977920.8253	190	Saudi Arabia	EU
Magellan Midstream Partners, LP	1409740.454	89	US	OGST

**Fig 9:**
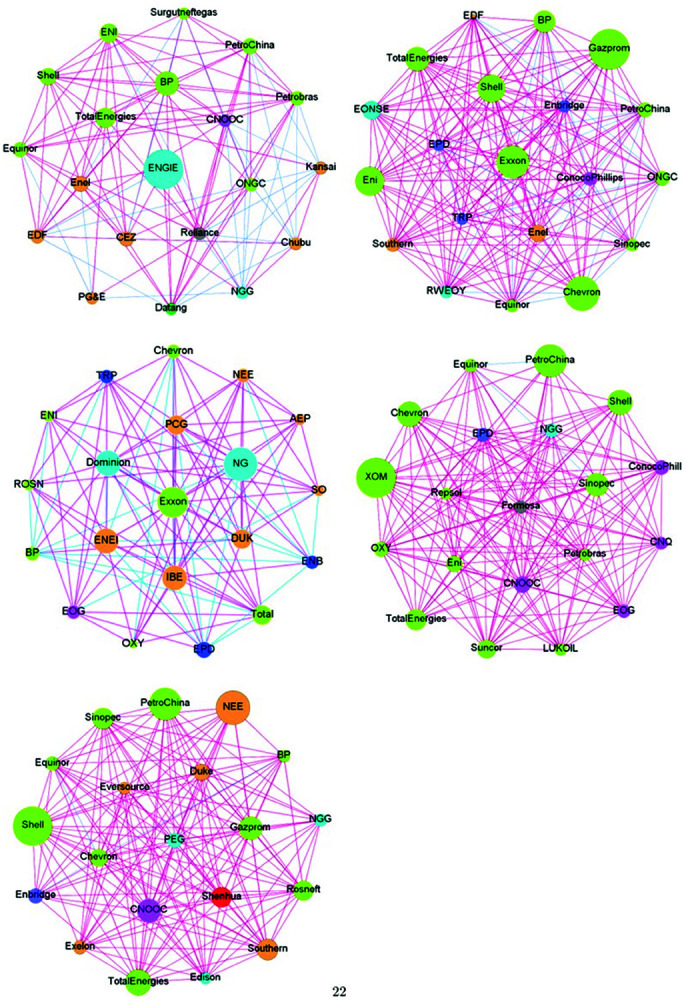
Shown in order from left to right, top to bottom, the risk connection networks among the top 20 energy companies with systemic risk rankings during global financial crisis, European debt crisis, Brexit Referendum, US–China trade conflict and COVID-19 pandemic.

Next, we conduct further analysis of the risk connection networks among energy companies under the five major shocks: the global financial crisis, the Eurozone debt crisis, the Brexit referendum, the US–China trade conflict, and the COVID-19 pandemic. During each period of these major shocks, we calculate the top 20 energy companies in terms of systemic risk scores for that period and construct the risk network among these companies using the breakpoint approach given by (2). The results are visualized in Fig 9, where energy companies are represented by colored nodes, with different colors indicating various industry categories. The size of the nodes corresponds to the magnitude of the systemic risk scores, with larger nodes signifying higher systemic risk. The lines connecting the nodes represent risk connections, with red lines indicating risk contagion (corresponding to the value of 1 in Eq 2) and blue lines indicating risk diversification (corresponding to the value of –1 in Eq 2).

Overall, there are close interactions in terms of risk, particularly strong comovements among energy companies that received high systemic risk scores during the five major shocks. Specifically, the risk networks were highly dense during the European debt crisis, the US–China trade disputes, and the COVID-19 pandemic period, while they were relatively sparser during the global financial crisis and the Brexit referendum. Moreover, within the context of the global financial crisis, a more pronounced risk diversification relationship emerged among energy companies. Notably, the top 20 ranked energy companies during this period primarily hailed from the Asia-Pacific region. Reflecting on the findings from Subsect 4.2.3, it suggests the possibility of a cascading relationship between companies in the Americas and those in Europe and Asia during this crisis. As risk transmission reached Europe and the Asia-Pacific region, it had largely dissipated, resulting in a comparatively lower severity of retransmission among companies in these regions, thereby contributing to a relatively sparser risk connection network. After this crisis, the increasing density of the risk networks reflects the growing financial integration among top energy companies, emphasizing the importance of analyzing risk interactions within the global energy market.

From an industry distribution perspective, among the top-ranked companies during several large shocks, the IOG industry has the highest number of companies, followed by the EU industry. At the company level, TotalEnergies SE, Royal Dutch Shell plc, PetroChina Co Ltd and Equinor ASA all have high systemic risk scores across the four large shocks outside of the Brexit Referendum and rank among the top companies for each of the four major shocks. These companies are traditional and influential oil companies from France, the Netherlands, China, and Norway. They hold significant market positions, and any crisis they face could potentially have a profound impact on the entire oil market and even the global energy market. Additionally, BP plc, Eni SpA, CNOOC Ltd, National Grid plc, Chevron Corp, and China Petroleum & Chemical Corp (We use the company’s abbreviation Sinopec in the network graph instead of China Petroleum & Chemical Corp) has also appeared multiple times in the network graphs. The proper management of these companies has a significant impact on the security of the energy market. Paying attention to the operational performance of these companies provides valuable insights for investors in the energy market, particularly during periods of significant risk.

As noted in the study by [4], not all companies that rank highly in Platts rankings are considered important nodes. During periods of large shocks, our research confirms this phenomenon. Companies that rank high in terms of systemic risk scores are a mixture of those with high and low Platts rankings. We also reference [3], which examines the influence of market capitalization on risk spillover among the world’s top 20 oil-related companies. We assess the collective impact of Platts rankings, market capitalization, and risk connections on systemic risk contributions, enabling a more comprehensive selection of globally influential companies within the energy market’s risk network.

### 4.3 External perspective: the risk formulation mechanism of the global energy market

The previous sections provide an analysis of the internal structure of risk connections within the global energy market. In this section, we further investigate how monthly returns, particularly the tail distribution of returns of top energy companies, are influenced by factors such as risk interconnectivity among companies, network node characteristics, and other common factors.

Reference [81] find that during crises, overall interconnectedness increases, leading to higher fragility in financial markets characterized by high levels of coordination, contagion, and spillover effects. Furthermore, considering the persistence of risk contagion, it implies that contemporaneous and lagged network effects are prevalent in the risk interconnections among global energy companies. In addition, [79] find that firm-specific characteristics play an important role in identifying systemically important non-financial firms, while [4] also suggest that market risk factors have a significant impact on total systemic risk. Therefore, we utilize the DNQR model proposed by [43] and summarized in Sect 3.3 to separately investigate the impacts of the risk network effects, firm-specific characteristics, and market environment factors on the stock returns of global top energy companies across different quantiles. In particular, the market environment factors include financial market-related factors and policy transmission effects.

Combining the ranking criteria of Platts top 250 global energy companies and the research by [79], we select total assets (Size), operating margin (OM), and debt-to-equity (DE) ratio as firm-specific characteristics, reflecting company size, profitability, and debt leverage, respectively. As for market environmental factors, we refer to [4] and chose WTI crude oil price (WTI), the crude oil volatility index (OVX), the US dollar price index (DXY), and the S&P 500 implied volatility index (VIX), which respectively represent the influences of the crude oil market, foreign exchange market, and investor sentiment on financial markets. We also include the Bloomberg Barclays Global Aggregate Index (BBGA), which reflects the impact of the bond market. Considering the frequent changes in economic policies, we also study the impact of economic policy uncertainty (EPU). Furthermore, compared to [82] and [42], who also use network quantile regression to study the influence of network effects on tail risks, our study employs the DNQR method, which takes into account the effects of contemporaneous network dynamics and allows for a rapid response to risk contagion among energy companies.

**Fig 10:**
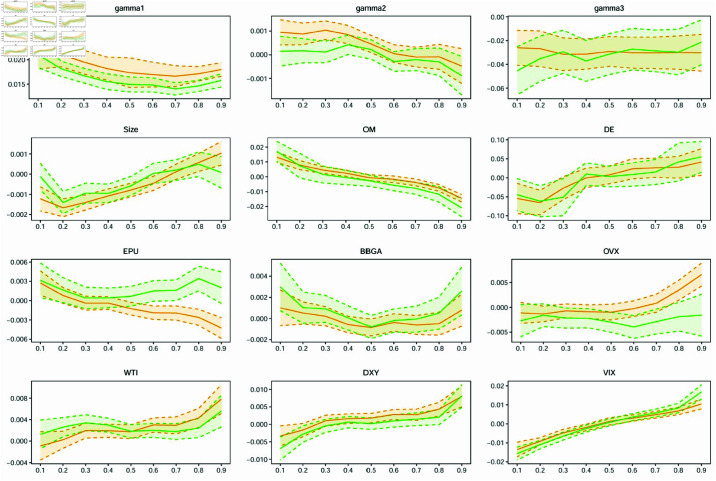
Coefficients of the quantile regression during five great shocks (green line) and the full sample period (yellow line). The colored region between the two dashed lines represents a kernel density-based confidence band, as advanced by [83].

Fig 10 presents the coefficients corresponding to different quantile levels of the quantile regression (4) during four large shocks (green line) and compares them with the coefficients from the full sample period (yellow line). The quantile levels (the x-axis of each plot in Fig 10) are set from 0.1 to 0.9 with a step size of 0.1. For each color, the colored region between the two dashed lines represents a kernel density-based confidence band, as advanced by [83]. The titles of the three plots in the first row of Fig 10, namely “gamma1”, “gamma2”, and “gamma3” represent $\gamma_{1}^{0}(\cdot )$, $\gamma_{2}^{0}(\cdot )$, and $\gamma_{3}^{0}(\cdot )$ in (4), respectively. As aforementioned, “gamma1” and “gamma2” measure the effects of contemporaneous and lagged network variables on the quantile of returns, respectively, while “gamma3” captures the temporal dynamic effects of the companies on themselves. Obviously, there are evident contemporaneous network effects among the world’s top energy companies. Especially when facing left-tail risks, the risk contagion effect within the energy company network becomes stronger and exhibits a downward trend with the quantile level. Furthermore, regarding “gamma2”, we find that the lagged network effects on left-tail returns significantly diminish or even disappear, while there might be reversal effects for right-tail returns. The reversal effects are more pronounced in the plot for “gamma3”, which shows significant negative dynamic effects of companies on themselves at various quantile levels. Our findings reflect that the extreme values of a particular energy company’s stock returns, especially extreme losses, are more likely to be driven by the market performance of other energy companies that exhibit systemic risk comovement during the same period. The DNQR model we employ effectively captures this influence.

Turning to the effects of firm-specific characteristics as shown by the three plots in the second row of Fig 10, in the entire sample period, we observe that the impact of firm size initially decreases in the left tail and then shows an upward trend as we move across different quantile levels. Furthermore, larger firm size has a more pronounced effect on left-tail risk, especially in the 10% to 20% quantile range. This suggests that firm size has a significant influence on the extreme risk of returns for energy companies. However, this effect is not significant during four large shock periods, possibly because energy companies, regardless of their size, cannot avoid exposure to risk shocks in such high-risk periods. Conversely, higher operating margins in energy companies are associated with lower left-tail risk, suggesting that a stronger operating margin offers greater protection to these companies during extreme shocks. On the flip side, energy companies with higher operating margins see a corresponding decrease in right-tail risk, leading to an overall reduction in tail risk for these companies. This effect becomes especially significant during periods of large shocks. Higher operating margins indicate greater profitability for companies, endowing them with a level of resilience against extreme losses and making them more appealing to investors, thereby boosting stock prices. As for the company’s debt level, the coefficient initially decreases in the left tail and then gradually increases with changing quantile levels. Although high leverage can reduce a company’s financial costs, it can also have adverse effects during times of crisis, such as decreased solvency, increased sensitivity to interest rate changes, cash flow instability, and high debt costs, exacerbating the left tail of energy companies.

In recent years, several studies have discovered that macroeconomic policy uncertainty significantly affects cross-asset correlations under various market conditions [84, 85]. Therefore, we extend our analysis to consider the influence of EPU. We observe that EPU exhibits a decreasing trend as the quantile level changes in the full sample period. However, during periods of large shocks, the impact of EPU tends to rise slowly and then decrease at higher percentile levels. We hypothesize that the instability of EPU’s impact during major shock periods is primarily due to the non-persistence of policy uncertainty, which triggers speculative behavior among market participants and leads to increased stock volatility for companies.

Lastly, we investigate the influence of financial market factors. We find that, apart from the bond index, other factors exhibit an increasing impact on the stock returns of energy companies as the quantile level increases. Specifically, we observe a U-shaped pattern in the impact of the bond price index during large shocks. Usually, the increase in BBGA reflects the overall prosperity of the global bond market and a relatively positive investor sentiment, as well as expectations of a looser monetary policy environment in the future. Under this market condition, it is not surprising that the left-tail risk of energy companies has decreased. Given the significant impact of debt factors during the European debt crisis and the COVID-19 pandemic, the influence of debt issues on the left-tail risk of energy companies deserves attention. For factors related to the crude oil market, both the changes in crude oil prices represented by the WTI index and investor sentiment in the oil market represented by the OVX show a stronger impact on right-tail risk during the entire sample period. However, during large shocks, the extreme impact of the oil market on stock returns is not particularly significant. This may be due to the fact that the oil market often experiences severe fluctuations in a short period during large shocks, and capturing this risk may require higher-frequency data. Furthermore, [4] find that WTI only became the primary driver of risk in the energy market in 2014 and 2015, while our study primarily focuses on global large shock events, excluding these two years. Therefore, the findings of [4] indirectly support our findings as well. In addition, the quantile regression results show that the influence of DXY and VIX increases as the quantile level rises, and this influence is more pronounced during large shocks. Regarding DXY, as the US dollar serves as a major pricing and trading currency in the oil market, the study by [86] found that changes in the value of the US dollar can affect oil prices. The appreciation of the US dollar may reduce international oil demand and trigger a decline in oil prices, while the study by [87] found that the depreciation of the US dollar may lead oil producers to increase oil prices or reduce oil supply. On the other hand, the impact of exchange rate policy uncertainty will change the investment behavior preferences of energy companies and increase their risk-taking, and the impact of exchange rate policy uncertainty on energy companies with better growth opportunities and governance structures is weaker [88]. However,a strengthening DXY would increase production costs for energy import companies, particularly for petroleum refining enterprises, thereby exacerbating their operational risks. However, it would be advantageous for oil exploration companies. This can explain the significant impact of DXY on the stock returns of energy companies in both the left and right tails. As for VIX, numerous studies have found it to be a major influencing factor in energy market volatility. Our study further indicates that during periods of large shocks, an increase in VIX significantly amplifies both left-tail and right-tail risks for energy companies, confirming that the VIX is an important driving factor affecting the stock returns of energy companies, particularly in terms of extreme returns.

### 4.4 Policy implications

This paper extend the research of [2, 4] to investigate the market performance of top global energy companies under large shocks. We delve into the mechanisms of risk generation in the energy market during major international risk events, providing valuable insights and contributions for regulatory authorities, energy company managers and investors in the financial market.

From the perspective of investors, how to reasonably allocate their asset portfolio in energy companies remains an important task. Since energy companies exhibit significant risk aggregation at the regional level and there are contemporaneous network effects in the energy market, therefore it is reasonable for investors to diversify their portfolios at the country and geographic levels, and it is risky to concentrate their energy investments in a single country. For example, during the 2022 Russia–Ukraine conflict, the United States and Europe imposed multiple sanctions on Russia, including export bans and energy price limitations. These sanctions led to a substantial series of changes in the dynamics of global energy trading due to Russia’s significant role in the market [89]. In addition, the results of the Systemic Risk Score show that the companies with the highest Systemic Risk Scores in several shocks are more likely to come from the Integrated Oil and Gas (IOG) and Electric Utilities (EU) sectors. For investors, portfolios should avoid being entirely concentrated in these industries.

From the perspective of energy companies, they are unable to allocate assets quickly and flexibly like investors. For example, China’s three largest energy companies, State Grid Corporation of China, China National Petroleum Corporation (CNPC), and China Petrochemical Corporation (Sinopec), ranked 2nd, 4th, and 5th respectively. These companies are tasked with securing China’s electricity supply, as well as the extraction of oil and natural gas, refining, and the production and marketing of chemical products. The existence of geographic clustering effects and contemporaneous network effects among energy companies require energy companies pay more attention to the individual characteristics of their own company and macroeconomic indicators. For example, compared to company size(Size), the company’s profitability (OM) is more helpful in resisting left tail risk. Excessive debt-to-equity (DE) ratio will weaken a firm’s performance in the face of left-tail risk, while higher DE cannot effectively improve the company’s own performance when facing right tail risk. At the macro level, economic policy uncertainty is actually a contrarian indicator when facing left-tail events. This implies that shifts in economic policy are important for the performance of energy companies in face of left-tail risk. For energy companies, due to the special position of the US dollar in commodity trading, especially in energy commodities, they also need to pay close watch on changes in the US dollar price index. In the energy sector, crude oil prices still have a good indicative role, with both OVX and WTI indicators showing that facing left-tail periods, the crude oil market will greatly influence the market performance of energy companies.

Finally, for energy policymakers, energy security may be even more important. Above all, geopolitical issues are inescapable to policymakers; large geopolitical conflicts involving energy suppliers can have a dramatic impact on energy networks. Reference [90] indicate that during the Russia–Ukraine war, oil exhibits strong spillover effects on financial assets, although these effects are temporary and dissipate over time. The best way to deal with such potential risks is to be proactive and enrich their energy import and export network without becoming overly reliant on a particular region or country. Secondly, when facing a large shock, the focus should not be on the size of the company, but on its role in the risk network. Energy companies ranked lower may also play a key role in risk contagion. Policymakers also need to realize that macroeconomic policies have significant impact for energy security, and even changing economic policy expectations can be helpful in face of large shocks, while policies that exacerbate volatility in market sentiment need to be more cautious. Also because of the aggregation of energy risks at the regional level, increased coordination of economic and energy policies among regional countries could be considered.For instance, considering the regional aggregation of energy risks, coordination of energy policies among countries within a region may be considered.

## 5 Conclusion

Amidst the frequent occurrences of major risk events, investigating the risk network of top global energy companies and the underlying causes is of paramount importance for safeguarding the stability and sustainable development of the international energy market. In this paper, we conduct an analysis from two perspectives: the internal risk correlation structure within the energy market and the external influencing factors on stock return risk for top global energy companies, in the context of four major shocks - the global financial crisis, the European debt crisis, the US–China trade disputes, and the COVID-19 pandemic. Our findings can be summarized in the following three aspects.

Firstly, we examine an investigation into the internal risk structure among the world’s top energy companies. Our findings reveal that the systemic risk levels of energy companies experience varying degrees of increase during huge shocks, experiencing significant surges during the global financial crisis and the COVID-19 pandemic. Further investigating the risk interconnections, we observe that during the global financial crisis, top energy companies exhibit high intensity of risk contagion on a global scale. During the period of the US–China trade disputes, energy companies demonstrate a phenomenon of cross-regional risk comovement, whereas during the COVID-19 pandemic, this risk comovement manifests as more pronounced regional effects. However, during the European debt crisis, the degree of risk interconnection among top energy companies is comparatively weaker. We also observe an extremely strong risk interconnection among top energy companies following the outbreak of the Russia–Ukrainian conflict. The combination of the conflict’s impact and the negative repercussions of the COVID-19 pandemic on the global energy market is expected to result in heightened fragility in the global energy market.

We conduct a detailed analysis of the systemic risk characteristics of Chinese energy companies during great shocks. Our findings indicate that Chinese energy companies exhibit relatively low systemic risk levels during large shocks, with their systemic risk primarily influenced by domestic and international energy market dynamics and macroeconomic policies. Furthermore, China’s top energy companies demonstrate extensive risk dependence with external counterparts, particularly with US energy companies, which has shown gradual strengthening of risk linkages over time. In contrast, the internal risk contagion within the Chinese energy market is relatively weak. Hence, it can be recognized that the primary threats to the security of China’s energy market originate from external factors rather than domestic ones.

Secondly, considering the risk interconnectedness and market capitalization levels of global top energy companies, we estimate systemic risk scores to identify PetroChina as a critical energy company. And we observe a close risk comovement relationship among companies with higher systemic risk scores during large shocks. Additionally, we confirm the significant positions of Royal Dutch Shell plc, TotalEnergies SE, Chevron Corp, and Exxon Mobil Corp within the risk network, as previously evidenced in [2, 4].

Finally, we employ a dynamic network quantile regression model to analyze the external factors influencing extreme risk for top energy companies. Our findings indicate that, under such large shocks, top energy companies are more susceptible to the driving effect of contemporaneous risk network interactions on extreme risk. Additionally, companies with higher operating profits are exposed to a lower level of extreme risk. Taking into consideration other pertinent factors from the financial market, we find that the impact of debt leverage on left-tail risk for energy companies deserves careful attention, while the crude oil market demonstrates a more pronounced influence on right-tail risk. Furthermore, foreign exchange market and investor sentiment contribute to simultaneous increases in both left and right-tail extreme risks for energy companies.

At the end, we would also like to recognize the limitations in our study. First, the EMES metrics we use to measure systemic risk is calculated by AdaBoost, although [60] verify that AdaBoost is the most superior when calculating EMES among multiple machine learning algorithms, the efficiency of other machine learning algorithms to study energy market risk relies on further research in the future. Second, our sample of firms comes from a set of firm rankings determined by a specific set of operating metrics, and the results when the sample firms change remain to be examined. In addition, while there are commonalities in the risk networks of energy firms under different large shocks, there are still differences in the characteristics of systemic risk from different types of shocks, and the mechanisms behind such differences have yet to be thoroughly investigated. Moreover, One limitation of our research is that although we found a strong risk resonance relationship among top energy companies after the outbreak of the Russia–Ukraine conflict, due to sample restrictions, our analysis of the Russia–Ukraine conflict was not sufficient, and the impact of the COVID-19 epidemic was not stripped away. As the most serious geopolitical event in recent decades, the Russia–Ukraine conflict has brought a huge impact on the global energy market, and the mechanism of the risk generation of the Russia–Ukraine conflict on the global energy market deserves to be further investigated. In the future, further discussion of the above limitations will enrich and deepen the study of systemic risk measurement and risk contagion in energy markets.

## Supporting information

S1_Appendix This table presents summary statistics of top energy companies in the Platts ranking investiaged in this paper. The Assets, Revenue, Profit, ROIC and CGR are financial indicators in 2021 from the Platts ranking. The maximum (Max), minimum (Min), mean (Mean, in percentage) and standard deviation (Std.) of energy companies’ stock returns are shown from January 1, 2007 to August 31, 2022
(PDF)

S1_Code and Data These materials containing data and code are used to reproduce the paper.
(PDF)
