# Supplementary material for: Risk formulation mechanism among top global energy companies under large shocks
Source: PLoS One. 2025 May 23;20(5):e0322462. doi: 10.1371/journal.pone.0322462 (PMC12101850; doi:10.1371/journal.pone.0322462)
Supplement: S1_Appendix — This table presents summary statistics of top energy companies in the Platts ranking investiaged in this paper. The Assets, Revenue, Profit, ROIC and CGR are financial indicators in 2021 from the Platts ranking. The maximum (Max), minimum (Min), mean (Mean, in percentage) and standard deviation (Std.) of energy companies’ stock returns are shown from January 1, 2007 to August 31, 2022 (PDF) [file pone.0322462.s001.pdf]

Table S1_Appendix .1 This table presents summary statistics of top energy companies in the Platts ranking investiaged in this paper. The Assets, Revenue, Profit, ROIC and CGR are financial indicators in 2021 from the Platts ranking. The maximum (Max), minimum (Min), mean (Mean, in percentage) and standard deviation (Std.) of energy companies' stock returns are shown from January 1, 2007 to August 31, 2022.

Rank	Name	Sector	Region	Country	Assets	Revenue	Profit	ROIC	CGR	Max	Min	Mean	Std
2	China Shenhua Energy Co Ltd	CCF	Asia/Pacific Rim	China	86854	35992	5531	0.07	-0.021	0.262	-0.268	0.0100	0.026
3	Reliance Industries Ltd	OGRM	Asia/Pacific Rim	India	177646	62781	5847	0.04	0.046	0.194	-0.178	0.0546	0.021
4	Fortum Oyj	EU	EMEA	Finland	68830	58358	2170	0.07	1.206	0.136	-0.142	-0.0186	0.019
5	Surgutneftegas Public JSC	IOG	EMEA	Russia	80372	14513	9454	0.13	-0.028	0.374	-0.224	-0.0021	0.025
6	Indian Oil Corp Ltd	OGRM	Asia/Pacific Rim	India	47721	48935	2909	0.10	-0.054	0.157	-0.206	0.0168	0.022
7	Enterprise Products Partners LP	OGST	Americas	US	64107	27200	3744	0.07	-0.024	0.219	-0.206	0.0151	0.018
8	Iberdrola, SA	EU	EMEA	Spain	145872	39463	4233	0.04	0.020	0.172	-0.152	0.0167	0.017
9	CNOOC Ltd	OGEP	Asia/Pacific Rim	China	111291	23973	3851	0.04	-0.059	0.202	-0.189	0.0119	0.025
10	China Petroleum & Chemical Corp	IOG	Asia/Pacific Rim	China	267521	324780	5107	0.03	-0.037	0.163	-0.180	-0.0096	0.022
11	The Southern Co	EU	Americas	US	122935	20375	3119	0.04	-0.040	0.172	-0.125	0.0186	0.013
12	Enel SpA	EU	EMEA	Italy	194610	76208	3108	0.02	-0.042	0.168	-0.221	-0.0098	0.018
13	Bharat Petroleum Corp Ltd	OGRM	Asia/Pacific Rim	India	21646	30947	2173	0.16	-0.008	0.164	-0.222	0.0483	0.024
14	TC Energy Corp	OGST	Americas	Canada	81089	10509	3603	0.05	-0.011	0.182	-0.247	0.0114	0.014
15	American Electric Power Co, Inc	EU	Americas	US	80757	14918	2200	0.04	-0.011	0.124	-0.123	0.0215	0.014
16	PetroChina Co Ltd	IOG	Asia/Pacific Rim	China	383911	298385	2933	0.01	-0.014	0.181	-0.162	-0.0280	0.022

18	National Grid plc	MU	EMEA	United Kingdom	93557	20571	2283	0.03	-0.010	0.153	-0.102	0.0109	0.014
19	NextEra Energy, Inc	EU	Americas	US	127684	17997	2919	0.03	0.016	0.130	-0.144	0.0462	0.015
20	NTPC Ltd	IPPET	Asia/Pacific Rim	India	53644	14996	1968	0.04	0.082	0.121	-0.150	0.0116	0.019
22	OMV Aktiengesellschaft	IOG	EMEA	Austria	58663	19705	1498	0.04	-0.065	0.187	-0.213	-0.0017	0.023
23	Exelon Corp	EU	Americas	US	129317	33039	1963	0.03	-0.005	0.165	-0.175	-0.0004	0.017
24	Public JSC Rosneft Oil Co	IOG	EMEA	Russia	209969	70063	2011	0.02	-0.031	0.381	-0.274	0.0199	0.024
25	Oil & Natural Gas Corp Ltd	IOG	Asia/Pacific Rim	India	67752	53353	1467	0.03	0.120	0.170	-0.181	-0.0014	0.023
26	Hindustan Petroleum Corp Ltd	OGRM	Asia/Pacific Rim	India	18039	31216	1434	0.13	0.020	0.163	-0.274	0.0353	0.026
27	Enbridge Inc	OGST	Americas	Canada	129577	31600	2412	0.02	-0.041	0.188	-0.180	0.0253	0.015
28	E.ON SE	MU	EMEA	Germany	113567	73371	1258	0.02	0.177	0.181	-0.122	-0.0321	0.019
29	Tokyo Electric Power Co Holdings, Incorporated	EU	Asia/Pacific Rim	Japan	109156	52955	1633	0.02	0.001	0.306	-0.323	-0.0520	0.031
30	SSE plc	EU	EMEA	United Kingdom	30053	9502	3061	0.13	-0.370	0.135	-0.130	0.0013	0.015
31	Public JSC Gazprom	IOG	EMEA	Russia	319429	86471	1831	0.01	-0.012	0.253	-0.363	-0.0061	0.024
32	Public Service Enterprise Group Incorporated	MU	Americas	US	50050	9603	1905	0.06	0.018	0.158	-0.119	0.0167	0.016
33	Korea Electric Power Corp	EU	Asia/Pacific Rim	South Korea	178588	51490	1751	0.01	-0.004	0.138	-0.146	-0.0193	0.019
34	Yanzhou Coal Mining Co Ltd	CCF	Asia/Pacific Rim	China	39949	33173	1099	0.04	0.124	0.263	-0.190	0.0472	0.033

36	Chubu Electric Power Co, Incorporated	EU	Asia/Pacific Rim	Japan	51326	26496	1329	0.03	0.010	0.127	-0.143	-0.0247	0.018
39	Sempra Energy	MU	Americas	US	66623	11370	1914	0.04	0.057	0.144	-0.188	0.0271	0.015
41	Petroleo Brasileiro SA - Petrobras	IOG	Americas	Brazil	197097	48334	1419	0.01	-0.021	0.205	-0.352	0.0284	0.030
42	Xcel Energy Inc	EU	Americas	US	53957	11526	1473	0.04	0.004	0.107	-0.136	0.0293	0.013
43	CLP Holdings Ltd	EU	Asia/Pacific Rim	China	30160	10248	1475	0.06	-0.047	0.154	-0.154	0.0042	0.011
44	DTE Energy Co	MU	Americas	US	45496	12177	1366	0.04	-0.012	0.122	-0.150	0.0288	0.014
45	PTT Plc	IOG	Asia/Pacific Rim	Thailand	80190	50924	1185	0.02	-0.068	0.136	-0.292	0.0160	0.020
46	RWE Aktiengesellschaft	MU	EMEA	Germany	73423	16409	922	0.03	-0.002	0.155	-0.190	-0.0191	0.021
47	Polskie Górnictwo Naftowe i Gazownictwo SA	IOG	EMEA	Poland	16546	10316	1932	0.15	0.030	0.165	-0.101	0.0104	0.021
48	Entergy Corp	EU	Americas	US	58239	10114	1388	0.04	-0.030	0.138	-0.171	0.0055	0.015
49	Duke Energy Corp	EU	Americas	US	162388	23453	1262	0.01	0.004	0.125	-0.122	0.0158	0.013
51	Dominion Energy, Inc	MU	Americas	US	95905	14172	1412	0.02	0.040	0.159	-0.131	0.0170	0.014
52	The Kansai Electric Power Co, Incorporated	EU	Asia/Pacific Rim	Japan	72894	27913	984	0.02	-0.004	0.163	-0.159	-0.0234	0.021
54	PJSC Tatneft	OGEP	EMEA	Russia	17282	9858	1416	0.12	0.019	0.329	-0.223	0.0403	0.027
57	Power Grid Corp of India Ltd	EU	Asia/Pacific Rim	India	34387	5330	1618	0.06	0.098	0.187	-0.190	-0.0046	0.017

58	PPL Corp	EU	Americas	US	48116	7607	1468	0.04	0.007	0.138	-0.147	-0.0038	0.016
59	WEC Energy Group, Inc	MU	Americas	US	37028	7242	1200	0.05	-0.018	0.124	-0.199	0.0370	0.013
60	Polski Koncern Naftowy ORLEN Spólka Akcyjna	OGRM	EMEA	Poland	22061	22681	725	0.04	-0.033	0.134	-0.122	0.0052	0.023
61	China Coal Energy Co Ltd	CCF	Asia/Pacific Rim	China	43463	21750	911	0.03	0.200	0.200	-0.218	0.0104	0.030
62	Eversource Energy	EU	Americas	US	46100	8904	1205	0.04	0.047	0.138	-0.137	0.0291	0.014
63	Centrais Elétricas Brasileiras SA - Eletrobrás	EU	Americas	Brazil	35723	5805	1265	0.05	0.059	0.401	-0.236	0.0364	0.031
64	Consolidated Edison, Inc	MU	Americas	US	62895	12246	1101	0.02	0.006	0.166	-0.141	0.0178	0.012
66	EDP - Energias de Portugal, SA	EU	EMEA	Portugal	51133	14821	953	0.03	-0.075	0.124	-0.180	0.0061	0.017
67	ENN Energy Holdings Ltd	GU	Asia/Pacific Rim	China	13893	11050	969	0.11	0.141	0.142	-0.155	0.0673	0.026
69	Electricité de France SA	EU	EMEA	France	364199	82190	361	0.00	0.021	0.148	-0.173	-0.0386	0.021
70	FirstEnergy Corp	EU	Americas	US	44464	10607	1003	0.03	-0.004	0.163	-0.235	-0.0108	0.017
71	China Gas Holdings Ltd	GU	Asia/Pacific Rim	China	14430	7666	1183	0.11	0.23	0.185	-0.225	0.0487	0.029
72	Osaka Gas Co, Ltd	GU	Asia/Pacific Rim	Japan	20881	12313	730	0.04	0.017	0.115	-0.109	0.0009	0.015
73	Neste Oyj	OGRM	EMEA	Finland	11686	12331	848	0.1	-0.041	0.213	-0.127	0.0470	0.023

74	China Resources Power Holdings Co Ltd	IPPET	Asia/Pacific Rim	China	33431	8955	976	0.04	-0.017	0.175	-0.196	0.0070	0.026
75	Edison International	EU	Americas	US	69372	13578	739	0.02	0.033	0.143	-0.143	0.0101	0.016
77	Saudi Electricity Co	EU	EMEA	Saudi Arabia	129453	18322	560	0.01	0.107	0.095	-0.104	0.0160	0.017
79	Tenaga Nasional Berhad	EU	Asia/Pacific Rim	Malaysia	43590	10565	863	0.03	-0.022	0.210	-0.163	0.0021	0.014
80	Ameren Corp	MU	Americas	US	32030	5540	871	0.04	-0.021	0.164	-0.191	0.0137	0.015
81	Snam S.p.A.	GU	EMEA	Italy	30569	3298	1311	0.05	0.030	0.095	-0.213	0.0119	0.014
82	Huaneng Power International, Inc	IPPET	Asia/Pacific Rim	China	67614	26144	431	0.01	0.035	0.204	-0.154	-0.0140	0.025
83	Kunlun Energy Co Ltd	GU	Asia/Pacific Rim	China	23914	16857	552	0.03	0.072	0.208	-0.205	0.0221	0.025
85	Fortis Inc	EU	Americas	Canada	44854	7224	977	0.03	0.025	0.101	-0.116	0.0171	0.012
86	Abu Dhabi National Energy Co PJSC	MU	EMEA	United Arab	50889	6530	1037	0.02	0.000	0.143	-0.121	-0.0032	0.029
87	AGL Energy Ltd	MU	Asia/Pacific Rim	Australia	11070	9151	764	0.09	-0.011	0.101	-0.182	-0.0184	0.015
89	Huadian Power International Corp Ltd	IPPET	Asia/Pacific Rim	China	36200	14002	645	0.02	0.047	0.150	-0.276	0.0020	0.028
90	GAIL (India) Ltd	GU	Asia/Pacific Rim	India	10943	7714	825	0.10	0.017	0.152	-0.168	0.0260	0.022
91	PJSC LUKOIL	IOG	EMEA	Russia	81957	75330	208	0.00	-0.025	0.239	-0.220	0.0215	0.022
93	CMS Energy Corp	MU	Americas	US	29666	6680	755	0.04	0.005	0.129	-0.131	0.0353	0.014

94	The Hong Kong & China Gas Co Ltd	GU	Asia/Pacific Rim	China	19350	5270	774	0.05	0.08	0.194	-0.194	0.0112	0.014
95	Idemitsu Kosan Co,Ltd	OGRM	Asia/Pacific Rim	Japan	35694	41129	315	0.01	0.069	0.127	-0.141	0.0060	0.022
96	Beijing Enterprises Holdings Ltd	GU	Asia/Pacific Rim	China	26371	8808	681	0.03	0.060	0.219	-0.160	0.0080	0.022
98	China Resources Gas Group Ltd	GU	Asia/Pacific Rim	China	12087	7193	663	0.09	0.119	0.739	-0.241	0.0701	0.029
99	NRG Energy, Inc	EU	Americas	US	14902	9093	510	0.05	0.001	0.258	-0.198	0.0099	0.025
100	Datang International Power Generation Co, Ltd	IPPET	Asia/Pacific Rim	China	43255	14753	469	0.01	0.043	0.208	-0.211	-0.0229	0.027
101	Tokyo Gas Co,Ltd	GU	Asia/Pacific Rim	Japan	24717	15933	447	0.02	-0.002	0.106	-0.124	-0.0056	0.016
102	Terna SpA	EU	EMEA	Italy	24563	2957	935	0.05	0.041	0.095	-0.162	0.0253	0.014
104	Emera Incorporated	EU	Americas	Canada	25251	4451	758	0.04	-0.040	0.094	-0.122	0.0253	0.011
106	VERBUND AG	EU	EMEA	Austria	14352	3859	752	0.08	0.036	0.184	-0.186	0.0217	0.022
108	Acciona, SA	EU	EMEA	Spain	21750	8645	453	0.03	-0.026	0.155	-0.192	0.0078	0.022
109	CPFL Energia SA	EU	Americas	Brazil	9802	6168	727	0.1	0.049	0.116	-0.186	0.0430	0.019
110	Kyushu Electric Power Co, Incorporated	EU	Asia/Pacific Rim	Japan	46276	19242	271	0.01	0.028	0.196	-0.144	-0.0350	0.020
111	ONEOK, Inc	OGST	Americas	US	23079	8542	612	0.03	-0.111	0.288	-0.474	0.0299	0.025
112	Tohoku Electric Power Co, Incorporated	EU	Asia/Pacific Rim	Japan	40357	20641	265	0.01	0.034	0.195	-0.238	-0.0386	0.021
113	UGI Corp	GU	Americas	US	13985	6559	532	0.05	0.023	0.139	-0.188	0.0196	0.016

114	A2A S.p.A.	MU	EMEA	Italy	14556	7976	436	0.04	0.058	0.126	-0.211	-0.0207	0.020
116	Evergy, Inc	EU	Americas	US	27115	4913	618	0.03	0.241	0.149	-0.175	0.0241	0.015
117	Companhia Energética de Minas Gerais	EU	Americas	Brazil	10795	5036	572	0.09	0.051	0.176	-0.279	0.0574	0.025
118	Veolia Environnement SA	MU	EMEA	France	54011	30968	129	0.00	0.016	0.167	-0.240	-0.0216	0.021
120	Alliant Energy Corp	EU	Americas	US	17710	3416	614	0.05	0.003	0.140	-0.102	0.0297	0.014
121	Pinnacle West Capital Corp	EU	Americas	US	20020	3587	551	0.04	0.002	0.122	-0.180	0.0097	0.014
122	Companhia Paranaense de Energia - COPEL	EU	Americas	Brazil	9339	3719	764	0.13	0.099	0.184	-0.211	0.0568	0.024
123	Red Eléctrica Corporación, S	EU	EMEA	Spain	15292	2433	740	0.06	0.006	0.136	-0.143	0.0208	0.015
124	Hera S.p.A.	MU	EMEA	Italy	13138	8846	360	0.04	0.078	0.142	-0.192	-0.0068	0.017
125	Atmos Energy Corp	GU	Americas	US	15359	2821	601	0.05	0.007	0.143	-0.138	0.0319	0.014
127	CEZ,a.s.	EU	EMEA	Czech Republic	32774	9781	254	0.01	0.017	0.199	-0.165	-0.0033	0.017
128	Algonquin Power & Utilities Corp	MU	Americas	Canada	13224	1677	774	0.07	0.033	0.167	-0.639	0.0159	0.020
131	Empresas Copec SA	OGRM	Americas	Chile	25174	18059	191	0.01	-0.039	0.115	-0.131	0.0119	0.017
133	Shenzhen Energy Group Co, Ltd	IPPET	Asia/Pacific Rim	China	17599	3156	575	0.04	0.096	0.096	-0.482	-0.0073	0.027
134	Manila Electric Co	EU	Asia/Pacific Rim	Philippines	8007	5648	335	0.14	-0.009	0.197	-0.293	0.0514	0.024

135	CK Infrastructure Holdings Ltd	EU	Asia/Pacific Rim	China	21556	981	943	0.05	0.039	0.091	-0.135	0.0178	0.015
136	Shanxi Coking Coal Energy Group Co ,Ltd	CCF	Asia/Pacific Rim	China	10895	5209	302	0.05	0.056	0.096	-0.736	0.0086	0.034
137	Formosa Petrochemical Corp	OGRM	Asia/Pacific Rim	China	13588	14814	265	0.02	-0.127	0.094	-0.112	0.0042	0.017
138	Magellan Midstream Partners, LP	OGST	Americas	US	8197	2428	817	0.11	-0.011	0.302	-0.262	0.0252	0.018
139	Energy Transfer LP	OGST	Americas	US	95144	38954	-647	-0.01	-0.013	0.292	-0.544	0.0105	0.027
140	MDU Resources Group, Inc	MU	Americas	US	8053	5533	391	0.07	0.076	0.214	-0.225	0.0042	0.019
142	ENGIE SA	MU	EMEA	France	182381	66378	-2051	-0.02	-0.022	0.223	-0.189	-0.0262	0.019
143	Chevron Corp	IOG	Americas	US	239790	94471	-5543	-0.03	-0.095	0.205	-0.250	0.0203	0.019
144	The Williams Companies, Inc	OGST	Americas	US	44165	7719	208	0.01	-0.013	0.296	-0.428	0.0123	0.027
145	BKW AG	EU	EMEA	Switzerland	10244	3270	393	0.07	0.076	0.187	-0.190	-0.0046	0.017
146	TotalEnergies SE	IOG	EMEA	France	266132	119704	-7242	-0.04	-0.071	0.141	-0.182	-0.0021	0.018
147	AltaGas Ltd	GU	Americas	Canada	17408	4517	393	0.03	0.298	0.163	-0.254	0.0024	0.018
149	Hokkaido Electric Power Co, Incorporated	EU	Asia/Pacific Rim	Japan	18067	6687	314	0.02	0.004	0.158	-0.128	-0.0473	0.020
150	The Chugoku Electric Power Co, Inc	EU	Asia/Pacific Rim	Japan	30555	11802	131	0.00	-0.002	0.114	-0.102	-0.0306	0.016
152	ACEA S.p.A.	MU	EMEA	Italy	11518	3870	339	0.04	0.064	0.171	-0.150	-0.0054	0.018

153	Royal Dutch Shell plc	IOG	EMEA	Netherlands	379268	180543	-21680	-0.08	-0.161	0.203	-0.192	-0.0004	0.018
154	Shanxi Lu'an Environmental Energy Development Co, Ltd	CCF	Asia/Pacific Rim	China	11943	4007	298	0.04	0.033	0.096	-0.669	-0.0005	0.035
155	Electric Power Development Co, Ltd	IPPET	Asia/Pacific Rim	Japan	25652	8206	201	0.01	0.02	0.159	-0.135	-0.0235	0.020
156	Power Assets Holdings Ltd	EU	Asia/Pacific Rim	China	12046	164	790	0.07	-0.037	0.100	-0.110	0.0120	0.012
157	Exxon Mobil Corp	IOG	Americas	US	332750	179784	-22440	-0.10	-0.088	0.159	-0.150	0.0065	0.017
158	PG&E Corp	EU	Americas	US	97856	18469	-1318	-0.02	0.025	0.557	-0.741	-0.0341	0.031
160	BP p.l.c.	IOG	EMEA	United Kingdom	267654	180094	-20306	-0.12	-0.089	0.195	-0.217	-0.0087	0.019
161	Canadian Natural Resources Ltd	OGEP	Americas	Canada	60858	13657	-352	-0.01	-0.009	0.204	-0.345	0.0225	0.027
164	Korea Gas Corp	GU	Asia/Pacific Rim	South Korea	31569	18316	-151	0.00	-0.021	0.153	-0.162	0.0021	0.023
165	Valero Energy Corp	OGRM	Americas	US	51774	60115	-1426	-0.04	-0.121	0.272	-0.223	0.0239	0.027
167	Lundin Energy AB (publ)	OGEP	EMEA	Sweden	6653	2564	384	0.17	0.089	0.281	-4.020	-0.0267	0.070
169	Naturgy Energy Group, SA	GU	EMEA	Spain	47083	18270	-442	-0.01	-0.129	0.144	-0.183	0.0023	0.018
170	Hawaiian Electric Industries, Inc	EU	Americas	US	15004	2580	198	0.04	0.003	0.134	-0.206	0.0090	0.015
171	Shenergy Co Ltd	IPPET	Asia/Pacific Rim	China	11773	3041	369	0.04	-0.153	0.096	-0.402	-0.0057	0.023
172	Equinor ASA	IOG	EMEA	Norway	121972	45753	-5510	-0.08	-0.091	0.127	-0.195	0.0212	0.020

173	Iren SpA	MU	EMEA	Italy	11375	4227	280	0.03	0.002	0.108	-0.163	-0.0126	0.020
174	The AES Corp	IPPET	Americas	US	34603	9660	43	0.00	-0.028	0.249	-0.190	0.0038	0.024
177	China Power International Development Ltd	IPPET	Asia/Pacific Rim	China	24062	4422	261	0.01	0.126	0.177	-0.189	0.0023	0.025
178	Eni S.p.A.	IOG	EMEA	Italy	130549	53503	-10281	-0.12	-0.128	0.157	-0.234	-0.0196	0.019
179	Enagás, SA	GU	EMEA	Spain	10726	1254	529	0.05	-0.082	0.138	-0.160	0.0002	0.015
180	Shan Xi Huayang Group New Energy Co,Ltd	CCF	Asia/Pacific Rim	China	8869	4811	232	0.04	0.035	0.096	-0.933	0.0052	0.038
184	COSCO SHIPPING Energy Transportation Co, Ltd	OGST	Asia/Pacific Rim	China	10177	2528	366	0.04	0.188	0.308	-0.289	-0.0091	0.035
185	Cheniere Energy, Inc	OGST	Americas	US	35697	9293	-85	0.00	0.181	0.523	-0.452	0.0442	0.042
188	ConocoPhillips	OGEP	Americas	US	62618	19216	-2701	-0.06	-0.137	0.225	-0.286	0.0189	0.023
189	Origin Energy Ltd	EU	Asia/Pacific Rim	Australia	18884	9941	62	0.00	-0.011	0.288	-0.171	-0.0066	0.020
190	Repsol, SA	IOG	EMEA	Spain	58700	33634	-3980	-0.09	-0.072	0.168	-0.171	-0.0066	0.021
191	Elia Group SA/NV	EU	EMEA	Belgium	18056	2631	298	0.02	0.398	0.159	-0.139	0.0412	0.013
194	Suncor Energy Inc	IOG	Americas	Canada	68409	19938	-3492	-0.08	-0.083	0.221	-0.231	-0.0018	0.025
195	MOL Hungarian Oil & Gas Co	IOG	EMEA	Hungary	18680	13605	-54	0.00	-0.010	0.140	-0.162	0.0006	0.021
196	Southwest Gas Holdings, Inc	GU	Americas	US	8736	3299	232	0.04	0.090	0.128	-0.161	0.0178	0.017
197	Pingdingshan Tianan Coal. Mining Co, Ltd	CCF	Asia/Pacific Rim	China	8258	3456	214	0.04	0.026	0.096	-0.297	0.0037	0.032

198	GS Holdings Corp	OGRM	Asia/Pacific Rim	South Korea	21518	13577	-216	-0.01	-0.003	0.140	-0.130	0.0116	0.022
199	The National Shipping Co of Saudi Arabia	OGST	EMEA	Saudi Arabia	5628	2238	419	0.08	0.116	0.095	-0.105	0.0109	0.020
200	ATCO Ltd	MU	Americas	Canada	17948	3189	204	0.01	-0.050	0.132	-0.187	0.0158	0.015
201	EVN AG	EU	EMEA	Austria	9960	2537	238	0.04	-0.016	0.103	-0.115	-0.0038	0.016
203	Centrica plc	MU	EMEA	United Kingdom	23828	17049	-381	-0.04	-0.241	0.155	-0.211	-0.0376	0.019
205	Rubis	GU	EMEA	France	5868	4646	214	0.04	-0.003	0.091	-0.125	0.0136	0.017
206	EOG Resources, Inc	OGEP	Americas	US	35805	9934	-605	-0.02	-0.042	0.185	-0.386	0.0366	0.027
207	Royal Vopak N.V.	OGST	EMEA	Netherlands	7760	1428	358	0.05	-0.031	0.129	-0.168	0.0046	0.017
208	Occidental Petroleum Corp	IOG	Americas	US	80064	17809	-14377	-0.25	0.125	0.290	-0.764	0.0117	0.030
211	Spic Dongfang Energy Corp	MU	Asia/Pacific Rim	China	14104	2062	196	0.03	0.030	0.097	-0.603	0.0149	0.034
212	The Tata Power Co Ltd	EU	Asia/Pacific Rim	India	13291	4366	135	0.01	0.066	0.209	-0.163	0.0379	0.025
213	Shanghai Electric Power Co Ltd	IPPET	Asia/Pacific Rim	China	19896	3734	117	0.01	0.083	0.097	-0.235	0.0184	0.027
216	Public Power Corp SA	EU	EMEA	Greece	16294	5536	42	0.00	-0.020	0.250	-0.278	-0.0206	0.036
217	Sichuan Chuantou Energy Co,Ltd	IPPET	Asia/Pacific Rim	China	6377	159	488	0.08	0.089	0.096	-0.801	0.0295	0.030
218	Guangxi Guiguan Electric PowerCo,Ltd	IPPET	Asia/Pacific Rim	China	6909	1385	339	0.05	-0.024	0.097	-0.360	0.0038	0.026

219	Hokuriku Electric Power Co	EU	Asia/Pacific Rim	Japan	14403	5772	62	0.00	0.024	0.134	-0.240	-0.0440	0.018
221	Northland Power Inc	IPPET	Americas	Canada	9216	1666	283	0.03	0.144	0.151	-0.146	0.0311	0.015
222	Inpex Corp	OGEP	Asia/Pacific Rim	Japan	41832	6960	-1008	-0.030	0.000	0.150	-0.154	-0.0101	0.025
223	Parkland Corp	OGRM	Americas	Canada	7352	11327	66	0.01	0.136	0.148	-0.236	0.0244	0.020
225	IDACORP, Inc	EU	Americas	US	7095	1351	237	0.05	0.000	0.145	-0.138	0.0262	0.014
226	Plains All American Pipeline, LP	OGST	Americas	US	24497	23290	-2790	-0.14	-0.039	0.331	-0.424	-0.0196	0.026
227	Pioneer Natural Resources Co	OGEP	Americas	US	19229	7026	-200	-0.01	0.099	0.186	-0.461	0.0476	0.029
228	APA Group	GU	Asia/Pacific Rim	Australia	12046	1950	239	0.02	0.037	0.190	-0.143	0.0298	0.016
229	First Gen Corp	IPPET	Asia/Pacific Rim	Philippines	5708	1830	259	0.05	0.023	0.116	-0.180	-0.0149	0.023
230	NiSource Inc	MU	Americas	US	22040	4682	-73	0.00	-0.013	0.160	-0.184	0.0286	0.016
231	Shikoku Electric Power Co, Incorporated	EU	Asia/Pacific Rim	Japan	12911	6492	27	0.00	-0.006	0.143	-0.139	-0.0330	0.020
232	CenterPoint Energy, Inc	MU	Americas	US	33471	7418	-767	-0.04	-0.083	0.160	-0.220	0.0156	0.017
233	Qatar Gas Transport Co Ltd (Nakilat) (QPSC)	OGST	EMEA	Qatar	8969	980	319	0.04	0.035	0.426	-0.127	0.0363	0.019
234	Guanghui Energy Co, Ltd	IOG	Asia/Pacific Rim	China	8351	2335	206	0.03	0.230	0.096	-0.599	0.0219	0.033
237	Black Hills Corp	MU	Americas	US	8089	1697	228	0.04	0.003	0.144	-0.198	0.0175	0.017
238	YTL Corp Berhad	MU	Asia/Pacific Rim	Malaysia	16796	4608	-45	0.00	0.092	0.172	-0.136	-0.0176	0.018

240	Pembina Pipeline Corp	OGST	Americas	Canada	25399	5014	-385	-0.02	0.047	0.298	-0.311	0.0270	0.019
241	Guangzhou Development Group Incorporated	IPPET	Asia/Pacific Rim	China	6697	4893	139	0.02	0.085	0.096	-0.126	0.0007	0.025
242	Galp Energia, SGPS, SA	IOG	EMEA	Portugal	14873	13550	-656	-0.06	-0.094	0.221	-0.181	0.0111	0.021
243	Electricity Generating Public Co Ltd	IPPET	Asia/Pacific Rim	Thailand	6759	1058	275	0.04	0.038	0.094	-0.157	0.0172	0.014
244	Sembcorp Industries Ltd	MU	Asia/Pacific Rim	Singapore	10073	4046	104	0.01	-0.155	0.196	-0.127	0.0078	0.020
245	S-Oil Corp	OGRM	Asia/Pacific Rim	South Korea	13794	14795	-677	-0.06	-0.070	0.182	-0.149	0.0105	0.023
247	First Philippine Holdings Corp	EU	Asia/Pacific Rim	Philippines	7912	2201	200	0.03	0.008	0.142	-0.197	-0.0020	0.020
249	New Jersey Resources Corp	GU	Americas	US	5570	1954	194	0.04	-0.049	0.173	-0.238	0.0256	0.017
250	Portland General Electric Co	EU	Americas	US	9069	2145	155	0.03	0.022	0.150	-0.176	0.0163	0.015
